# IL4 receptor targeting enables nab-paclitaxel to enhance reprogramming of M2-type macrophages into M1-like phenotype via ROS-HMGB1-TLR4 axis and inhibition of tumor growth and metastasis

**DOI:** 10.7150/thno.92672

**Published:** 2024-04-08

**Authors:** Sri Murugan Poongkavithai Vadevoo, Yeoul Kang, Gowri Rangaswamy Gunassekaran, Seok-Min Lee, Min-Sung Park, Dong Gyun Jo, Sang-Kyun Kim, Ho Lee, Won Jong Kim, Byungheon Lee

**Affiliations:** 1 Department of Biochemistry and Cell Biology, School of Medicine, Kyungpook National University, 680 Gukchaebosang-ro, Jung-gu, Daegu 41944, Republic of Korea.; 2 Department of Biomedical Science, School of Medicine, Kyungpook National University, 680 Gukchaebosang-ro, Jung-gu, Daegu 41944, Republic of Korea.; 3 CMRI, School of Medicine, Kyungpook National University, 680 Gukchaebosang-ro, Jung-gu, Daegu 41944, Republic of Korea.; 4 Department of Chemistry, POSTECH-CATHOLIC Biomedical Engineering Institute, Pohang University of Science and Technology (POSTECH), 77 Cheongam-ro, Nam-gu, Pohang, Gyeongbuk 37673, Republic of Korea.; 5 Laboratory Animal Center, K-Medi Hub, 88 Dongnae-ro, Dong-gu, Daegu 41061, Republic of Korea.; 6 Laboratory Animal Research Facility, National Cancer Center, 323 Ilsan-ro, Ilsandong-gu, Koyang, Kyunggi 10408, Republic of Korea.

**Keywords:** IL4 receptor, M2-macrophage, nab-paclitaxel, reprogramming, immunotherapy

## Abstract

**Rationale:** Nab-paclitaxel (Abx) is widely employed in malignant tumor therapy. In tumor cells and pro-tumoral M2-type macrophages, the IL4 receptor (IL4R) is upregulated. This study aimed to elucidate the selective delivery of Abx to M2-type macrophages by targeting IL4R and reprogramming them into an anti-tumoral M1-type.

**Methods:** Abx was conjugated with the IL4R-binding IL4RPep-1 peptide using click chemistry (IL4R-Abx). Cellular internalization, macrophage reprogramming and signal pathways, and tumor growth and metastasis by IL4R-Abx were examined.

**Results:** IL4R-Abx was internalized into M2 macrophages more efficiently compared to the unmodified Abx and control peptide-conjugated Abx (Ctrl-Abx), which was primarily inhibited using an anti-IL4R antibody and a receptor-mediated endocytosis inhibitor compared with a macropinocytosis inhibitor. IL4R-Abx reprogrammed the M2-type macrophages into M1-like phenotype and increased reactive oxygen species (ROS) levels and extracellular release of high mobility group box 1 (HMGB1) in M2 macrophages at higher levels than Abx and Ctrl-Abx. The conditioned medium of IL4R-Abx-treated M2 macrophages skewed M2 macrophages into the M1-like phenotype, in which an anti-HMGB1 antibody and a toll-like receptor 4 (TLR4) inhibitor induced a blockade. IL4R-Abx accumulated at tumors, heightened immune-stimulatory cells while reducing immune-suppressing cells, and hampered tumor growth and metastasis in mice more efficiently than Abx and Ctrl-Abx*.*

**Conclusions:** These results indicate that IL4R-targeting allows enhancement of M2-macrophage shaping into M1-like phenotype by Abx through the ROS-HMGB1-TLR4 axis, improvement of antitumor immunity, and thereby inhibition of tumor growth and metastasis, presenting a new approach to cancer immunotherapy.

## Introduction

There is an abundance of tumor-associated macrophages (TAMs) in the tumor microenvironment. Majority of TAMs feature a pro-tumoral M2 phenotype, secreting immune-suppressive cytokines, such as transforming growth factor-β and IL10, and recruiting immune-suppressive cells, including myeloid-derived suppressor cells (MDSCs) and regulatory Treg cells 1-3. Furthermore, in tumor tissues, an increase in M2-type TAM population is a marker of poor cancer prognosis 4. Thus, M2-type macrophages in tumor tissues represent a promising target for cancer therapy.

An albumin-bound paclitaxel nanoparticle called nab-paclitaxel (trade name Abraxane, hereafter Abx) has been used in treating metastatic breast tumor, lung tumor, and pancreatic tumor 5. Paclitaxel is an antineoplastic agent that binds to microtubule tubulin and inhibits microtubule disassembly during mitosis, leading to cell cycle arrest and cancer cell apoptosis. In addition to tumor cell cytotoxicity, paclitaxel has been reported to activate macrophages into pro-inflammatory phenotype by binding to toll-like receptor 4 (TLR4) on the macrophage cell surface 6 or by binding to the TLR4 accessory protein, myeloid differentiation factor 2, in the cytoplasm 7. Abx is internalized into macrophages via macropinocytosis and drives pro-inflammatory M1 macrophage polarization that demonstrates antitumor activity 8. These findings indicate the potential of Abx as an immunotherapeutic agent that reprograms M2-type macrophages into the M1 phenotype in the tumor microenvironment and as a cytotoxic agent against tumor cells. However, the macrophage activation mechanism by Abx remains poorly explored.

In certain types of immune cells such as Th2 T-cells, the IL4 receptor (IL4R) comprising of IL4Rα and IL2Rγc subunits (named type 1 IL4R) is expressed 9, 10. Conversely, IL4R consisting of IL4Rα and IL13Rα subunits (named type II IL4R) is highly upregulated in certain types of tumors such as breast, lung, and pancreatic cancers, which is associated with poor prognosis 11-13. IL4 binds to the IL4Rα subunit of IL4R and eventually recruits the IL2Rγc or IL13Rα subunit 10. To our interest, IL4R is expressed in M2 macrophages at higher levels than in M1 macrophages 14-17. IL4 binding to IL4R induces M2 macrophage polarization 18. Additionally, IL4 and IL4R interaction enhances the pro-tumorigenic M2-macrophage activity and cathepsin protease production by TAMs 19, 20, which contributes to tumor progression and metastasis. These findings indicate that IL4R is a promising cell surface receptor to target M2 macrophages in the tumor microenvironment.

Selective nanoparticle delivery to tumors using ligands such as antibodies and peptides specific to molecules overexpressed in the tumor cell surface empowers nanoparticles to differentiate tumor cells from normal cells. Peptides have a lower affinity and shorter circulating time and are more vulnerable to degradation compared to antibodies. In contrast, peptides have a deeper tissue penetration ability, more efficient internalization into cells, and lesser immune reaction possibility 21-24. We have identified the IL4RPep-1 peptide consisting of CRKRLDRNC amino acid sequence using phage display, which is homologous to the KRLDRN sequence of IL4 and binds to IL4R 25. IL4RPep-1 has been successfully used as a tumor-homing peptide for the selective delivery of nanoparticles and liposomes carrying chemotherapeutic drugs or siRNA to tumors 26-28. This study aimed to enhance Abx delivery to IL4R-high M2-type macrophages via the surface modification of Abx with IL4RPep-1 and reprogramming of M2-type macrophages into M1-like phenotype for cancer immunotherapy. Furthermore, the pathways included in the reprogramming of M2 macrophages through the IL4R-targeted Abx were examined.

## Materials and Methods

### Cell cultures

4T1 mouse breast tumor cells were cultured in Roswell Park Memorial Institute media supplemented with 10% fetal bovine serum (FBS, Thermo Fisher, Waltham, MA). Lewis lung carcinoma (LLC) mouse lung tumor cells and Panc-1 human pancreatic tumor cells were cultured in Dulbecco's modified Eagle's medium (DMEM, HyClone, South Logan, UT) supplemented with 10% FBS. Cell cultures were maintained at 37 °C in a humidified 5% CO_2_ atmosphere.

### Bone marrow-derived monocyte (BMDM) preparation and polarization to M2-type macrophages

To differentiate into BMDMs, bone marrow cells were isolated from tibias and femurs of BALB/c mice and incubated with DMEM supplemented with 10 ng/mL of macrophage-colony-stimulating factor (Gibco, Carlsbad, CA) and 10% FBS for 7 days. The culture medium was altered every 2 to 3 days. For M2-macrohage polarization, BMDMs were incubated with 20 ng/mL of recombinant mouse IL-4 for 24-48 h.

### Peptide synthesis

IL4RPep-1 (sequence: CRKRLDRNC) and control peptide (sequence: CNSSSVDK) were synthesized and purified using high-performance liquid chromatography to >90% purity by Peptron Inc. (Daejeon, Korea). To prepare fluorescent peptides, peptides were conjugated at the N-terminus with fluorescein isothiocyanate (FITC) or Flamma 675 near-infrared fluorescence dye (BioActs, Incheon, Korea) during synthesis. The amino acid sequence of the control peptide came from the phage coat protein, in which random peptide sequences were inserted to construct a phage-displayed peptide library.

### Preparation of peptide-conjugated Abx

A total of 30 mg of Abx (containing 27 mg (or 406 nmol) of Albumin) was dissolved in 3 mL phosphate buffer saline (PBS) and mixed with 2.534 mg (487 nmol; 1.2 equivalent to Abx) of 1,8-bismaleimido-diethyleneglycol (BM(PEG)_2_, Thermo Fisher) and stirred for 1 h. After incubation, to eliminate unbound BM(PEG)_2_, the Abx-BM(PEG)2 conjugate was subjected to ultracentrifugation via an Amicon Ultra 30 kDa-4 mL centrifugal filter (Merck, Burlington, MA). To reduce the peptide disulfide bonds, each peptide (487 nmol; 1.2 equivalent to Abx) was incubated with 5 mM Tris-(2-carboxyethyl)phosphine hydrochloride (Sigma-Aldrich, St. Louis, MO) in 0.1-M Tris-HCl buffer for 10 min. The Abx-BM(PEG)_2_ was added to the reduced peptide solution and stirred for 24 h. Using the Amicon Ultra 30 kDa-4 mL centrifugal filter, the Abx-peptide conjugate was ultracentrifuged followed by freeze-drying. Thereafter, using the PD-10 desalting column and Sephadex G-25 resin (GE healthcare, Chicago, IL), the Abx-peptide conjugate was purified and was obtained as a yellowish powder.

### Characterization of peptide-conjugated Abx

The molecular weight of Abx-BM(PEG)_2_ and IL4R-Abx was determined using matrix-assisted laser desorption-ionization/time of flight mass spectrometry (MALDI-TOF-MS). MALDI-TOF-MS analyses were conducted using an Autoflex speed LRF, Bruker (smart beam-II laser (355 nm)) operating in linear mode/positive ion mode with a mass-to-charge (m/z) ratio range of 30-210 kDa. The laser fluence was set at 90%, with a laser frequency of 2000 Hz, and more than 10000 laser shots accumulated. The sample (4 mg/mL) was dissolved in an aqueous solution containing 50% acetonitrile (ACN) with 0.1% TFA (trifluoroacetic acid). Sinapic acid was served as the matrix. The hydrodynamic size of nanoparticle was measured by dynamic light scattering (DLS) with Zetasizer Nano S90 systems (Malvern, UK). All groups were dissolved at a concentration of 10 mg/mL in PBS buffer. To examine structural feature, transmission electron microscopy (TEM) images were obtained using JEM-1011 instrument (JEOL Ltd, Japan), and analyzed with Gatan digital microscope software. All groups are dissolved at a concentration of 0.01 mg/mL in deionized water.

### Internalization assays

Abx was labeled with FITC using fluorescein-EX protein labeling kit (Thermo Fisher). A 0.5 mL solution of 2 mg/mL abraxane was mixed with 50 µl of 1 M bicarbonate and incubated with the reactive dye for 1 h at room temperature. The labeled Abx was purified from free dye using a spin column (MW > 20 kDa). Cells (1 × 10^5^ cells/well of a four-well chamber) were incubated with 1% bovine serum albumin at 24°C for 30 min and then with 5 μg/mL of FITC-labeled Abx at 37 °C for 1 h. Cells were fixed with 4% paraformaldehyde followed by nuclear staining with 4', 6-diamidino-2-phenylindole (DAPI) after washing and observed under a confocal microscope (Nanoscope, Daejeon, Korea), or cells were analyzed using Attune NxT flow cytometer (Thermo Fisher). For the internalization inhibition, cells were incubated with 50 μM of 5-(n-ethyl-n-isopropyl) amiloride (EIPA, Thermo Fisher), 50 μM of chlorpromazine (CPZ, Sigma-Aldrich), and 100 μM of anti-IL4Rα blocking antibody or anti-IgG antibody (R&D Systems, Minneapolis, MN) for 30 min prior to incubation with FITC-labeled Abx.

### Cytotoxicity assays

Cells (5 × 10^3^ cells/well in a 96-well plate) were incubated with IL4R-targeted and unmodified Abx for 24 h at 37°C. Cells were incubated with a fresh culture medium containing 10% CCK-8 reagents (Dojindo, Kumamoto, Japan) for 1-4 h after treatments. Absorbance was measured at 450 nm. GraphPad Prism 7 software (GraphPad, New York, NY) was used to calculate the half maximal inhibitory concentration (IC50).

### Quantitative reverse transcription-polymerase chain reaction (qRT-PCR)

Using a miRNeasy mini kit (Qiagen, Hilden, Germany), RNA was isolated from the M2 macrophages and subjected to qRT-PCR. Primers for *arg1, il-10, il-4, tgf-β, il-12p40, ifn-γ*, and *β-actin* were obtained from Bioneer Inc. (Daejeon, Korea). cDNA was synthesized using PrimerScript 1st strand cDNA Synthesis Kit (Takara, Shiga, Japan). qPCR using SYBRGreen (Qiagen) was conducted on a real time cycler (Bio-Rad, Hercules, CA). According to the Livak method 29, data for relative expression were analyzed.

### Measurement of reactive oxygen species (ROS) levels and inhibition of ROS, high mobility group box 1 (HMGB1), and TLR4

Cells were incubated with IL4R-targeted and untargeted Abx for 6 h and then with 1 μM of 2',7'-dichlorodihydrofluorescein diacetate (H_2_DCFDA, Thermo Fisher) at 37 °C for 1 h, which is a cell-permeable ROS indicator and generates fluorescence through ROS. The fluorescence levels were observed under a fluorescence microscope (Nikon, Tokyo, Japan).Cells were incubated with 50 mM of N-acetyl-l-cysteine (NAC) for 30 min before treatment with IL4R-Abx and Abx to inhibit or block ROS activity. To inhibit or block HMGB1 and TLR4 activity, cells were incubated for 30 min with 10 μg/mL of anti-HMGB1 antibody (Arigo Biolaboratories, Hsinchu, Taiwan) and 500 nM of CLI095 (Thermo Fisher), respectively, before treatments with a conditioned medium (CM) of IL4R-Abx- and Abx-treated M2 macrophages.

### Analysis of intracellular and extracellular HMGB1

To examine the location of intracellular HMGB1, M2 macrophage cells were seeded at a density of 1 × 10^5^ cells in an 8-well chamber (Thermo Fisher) and allowed to attach overnight. Cells were incubated with Abx and IL4R-Abx (5 μg/mL) at 37 °C for 24 h. After incubation, cells were fixed with 4% PFA, permeabilized with 0.1% Triton X100, and incubated with an anti-HMGB1 antibody (Abcam, 1/400 dilution) at 4 °C overnight. Cells were then incubated with Alexa Fluor 647-labeled secondary antibody in dark at room temperature for 1 h. Cells were incubate with DAPI for nuclear staining and observed under a confocal scanning microscope (Nanoscope).

To examine the levels of extracellular HMGB1 released into the culture medium using western blotting, the culture medium of M2 macrophages treated for 24 h was collected and concentrated using a centrifugal concentrator (Vivaproducts, Littleton, MA). Samples were electrophoresed in 10% polyacrylamide gel and transferred to a polyvinylidene difluoride membrane. The membrane was blocked with 5% skimmed milk in Tris-buffered saline containing 0.1% Tween-20 and then incubated with antibodies against HMGB1 (Abcam, Cambridge, UK) and β-actin (Santa Cruz Biotechnology, Dallas, TX) at 4 ^ο^C overnight followed by incubation with a horse radish peroxidase-conjugated secondary antibody (Cell Signaling Technology, Danvers, MA) at room temperature for 1 h. A substrate (Thermo Fisher) was used to incubate the membrane, and a chemiluminescence detection reagent (FujiFilm, Tokyo, Japan) was used for visualization. The protein band intensity was quantified using ImageJ software (National Institute of Health (NIH), Bethesda, MD).

To measure the concentrations of HMGB1 in the culture medium using ELISA, 100 μL of the samples were added to each well of a 96-well plate coated with anti-mouse HMGB1 antibody and incubated at 37 °C for 2 h. After incubation, 100 μL of biotin-labeled anti-mouse HMGB1 antibody was added and incubated at 37 °C for 1 h followed by incubation with streptavidin-horseradish peroxidase conjugate at 37 °C for 1 h. The plate was incubated with tetramethylbenzidine substrate at room temperature in dark for 20 min, and the reaction was terminated by addition of 50 μL of stop solution. The absorbance at 450 nm was measured with a microplate reader (Thermo Fisher).

### Paclitaxel concentrations in tissues

Approximately 3 μL of each of standard paclitaxel solutions in acetonitrile was mixed with 27 μL of plasma and added to 270 μL of acetonitrile containing an internal standard solution (gliclazide at 100 ng/mL concentration). Tumor and control organs were dissected at 6 h after injection of Abx, added with cold PBS at a weight ratio of 1:4, and homogenized using a high-speed homogenizer. The supernatants acquired after homogenate centrifugation were used as sample solutions. The 30-μL sample solutions were added to 270 μL of acetonitrile containing the internal standard solution. A 5-μL aliquot of the sample and paclitaxel standard solutions were injected into the ultraperformance liquid chromatography-mass spectrometry/mass spectrometry system (Agilent, Santa Clara, CA). The paclitaxel concentrations in samples were calculated using TargetLynx software (Waters, Milford, MA).

### Immune cell population analysis in tissues

Tumors were excised and fragmented into several pieces to prepare the tumor-infiltrating leukocytes. The fragmented tissues were further minced into 2-3 mm^3^ pieces and incubated with collagenase D (Roche, Basel, Switzerland) at 37 °C for 40 min. The tissue samples were filtered through a 100-µm cell strainer. Dead cells and cellular debris were removed using Ficoll (GE Healthcare, Buckinghamshire, UK) gradient centrifugation. Collected cells were re-suspended in PBS containing 2% FBS. Utilizing a flow cytometer, cells were gated upon CD45 expression. Cells were incubated for 20 min in the dark with fluorescently labeled antibodies against CD11b, CD3, CD4, CD8, CD45, F4/80, MHCII, Foxp3, and Ly6C (BioLegend, San Diego, CA) for surface biomarker staining. Cells were analyzed using Attune NxT flow cytometer (Thermo Fisher).

### Animal models

Six- to 8-week old mice were purchased from Orient Bio (Seongnam, Korea). Animal care was conducted in accordance with the Guidelines of the Institutional Animal Care and Use Committee of Kyungpook National University (permission No. 2015-0017). BALB/c mice were injected with 1×10^6^ 4T1 cells in the lower right mammary fat pad to establish 4T1 orthotopic breast tumor. To establish subcutaneous LLC lung tumor and Panc-1 pancreatic tumor models, C57BL/6 mice and BALB/c nude mice were injected with 5×10^5^ LLC and Panc-1 cells in the lower right flank, respectively. *K-ras^LA2^* mutant transgenic *mice* provided by the NIH (Bethesda, MD) were mated with wild-type (WT) C57BL/6N mice to establish a spontaneous lung tumor. For genotyping, complementary DNA was purified through QIAquick columns (Qiagen) and amplified using PCR with an annealing temperature of 60°C for 35 rounds using PCR primers as previously described 30. KPC (*LSL-Kras^G12D/+^LSL-p53^R172H/+^PDX-1 Cre*) transgenic pancreatic tumor mice were obtained through cross breeding *LSL-Kras^G12D/+^ PDX-1 Cre* with* LSL-p53^R172H/+^PDX-1 Cre*. For genotyping, PCR primers used were as follows: PDX-1 Cre (F), CTG GAC TAC ATC TTG AGT TGC; PDX-1 Cre (R), TTC TTG CGA ACC TCA TCA CTC GTT G; K-ras WT (F), TGT CTT TCC CCA GCA CAG T; K-ras Com (R), CTG CAT AGT ACG CTA TAC CCT GT; K-ras Mut (F), GCA GGT CGA GGG ACC TAA TA; P53 KI WT, AGG TGT GGC TTC TGG CTT C; P53 KI Com, GAA ACT TTT CAC AAG AAC CAG ATC A; P53 KI Mut, CCA TGG CTT GAG TAA GTC TGC A.

### Whole-body and *ex vivo* fluorescence imaging

Through the tail vein, mice were intravenously injected with 100 µL (at 5 μg/mL) of targeted and untargeted Abx conjugated with peptides labeled with Flamma 675 near-infra red fluorescent dye (BioActs) at the N-terminus during synthesis. After a 6-h circulation, mice were anesthetized, and *in vivo* images were taken using the IVIS imaging system (PerkinElmer, Waltham, MA). After *in vivo* imaging, the mice were sacrificed, and the tumors and other organs were isolated and subjected to *ex vivo* imaging using the IVIS imaging system, and the total flux (photon/second) was measured using Living Image 4.5 software (PerkinElmer).

### Histological analysis

Frozen tissue sections (8 µm thickness) were stained at 37 ^o^C for 1 h using anti-mouse IL4Rα (Santa Cruz Biotechnologies). Alexa594-conjugated antibody (Life technologies) was used as a secondary antibody. Tissue samples were counter-stained with DAPI, incubated with Prolong Gold^®^ anti-fade mounting reagent (Life technologies), and observed under a confocal microscope (Nanoscope).

### Antitumor therapy

4T1, LLC, and Panc-1 tumor-bearing mice were subjected to randomization and grouping for initiation of therapy at 2 - 3 weeks after tumor inoculation when the tumor sizes reached approximately 100 mm^3^. *K-ras^LA2^* mutant mice and KPC mice were used for treatments at 10 weeks of age. Targeted and untargeted Abx was injected intravenously via the tail vein of mice (5 or 10 mg/kg body weight, weekly for 4 weeks). In 4T1, LLC, and Panc-1 tumors, tumor size was measured using a caliper during treatments. At the treatment completion, mice were sacrificed and tumors and lungs were isolated, weighed, and assessed for metastatic tumor masses. Additionally, tumors were subjected to analysis of immune cell populations using flow cytometry or immunohistochemistry. At the end of treatments, mouse blood was collected and analyzed for liver function, such as aspartate transaminase (AST) and alanine transaminase (ALT), and kidney function, such as blood urea nitrogen (BUN) and creatinine (CRE), by K-Medi Hub (Daegu, Korea).

### Statistical analysis

Data are presented as the mean ± standard deviation (SD). GraphPad Prism 7 software was used for all statistical analyses. Statistical significance was determined using one-way analysis of variance (ANOVA) followed by Tukey's multiple post hoc test or two-way ANOVA followed by Bonferroni multiple post hoc test. *, *P* < 0.05; ** *P* < 0.01; ***, *P* < 0.001.

## Results

### Preparation and characterization of IL4R-targeted Abx

**Figure 1:**
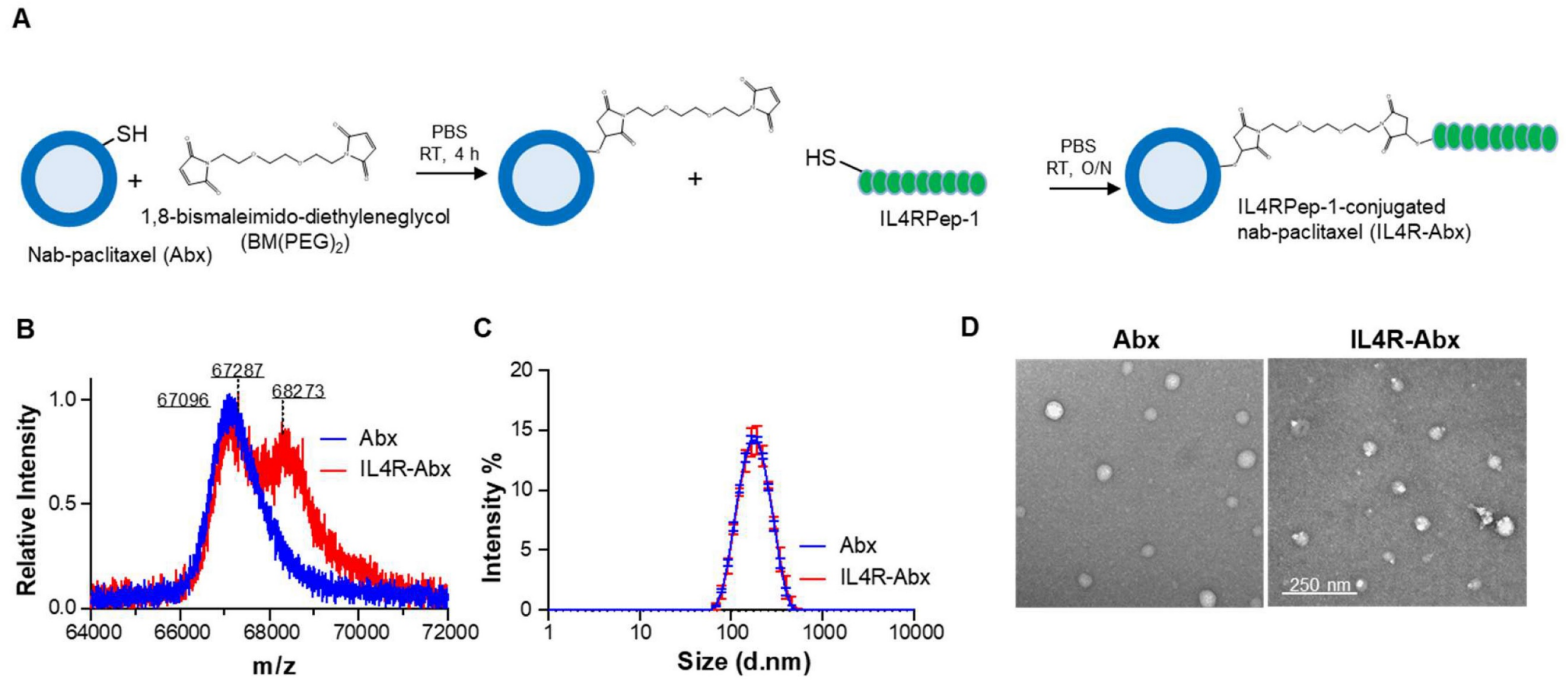
Preparation and characterization of IL4R-Abx. **(A)** Synthetic schemes of IL4RPep-1-conjugated Abx (IL4R-Abx). **(B)** MALDI-TOF-MS spectra of albumin part (64-72 kDa) in Abx (theoretical MW_albumin_ = 66348 g/mol) and IL4R-Abx. **(C)** Size distribution of Abx and IL4R-Abx by DLS. **(D)** TEM image of Abx and IL4R-Abx. Scale bars, 250 nm.

To confer an IL4R-targeting activity to Abx, a sulfur-group-containing Abx on the albumin surface was conjugated with a bismaleimide linker and then conjugated to a sulfur group of cysteine residue of IL4RPep-1 (CRKRLDRNC, MW = 1,163 g/mol) using the same linker and called the IL4R-Abx (Figure 1A). The increase in molecular weight of albumin resulting from the connection of the free thiol group of albumin and the cysteine residue of the IL4RPep-1 using BM(PEG)_2_ was measured by MALDI-TOF-MS spectrometry. This increase was observed from 67,096 g/mol (theoretical MW = 66,348 g/mol 31) to 67,287 g/mol, and subsequently to 68,273 g/mol (Figure 1B and S1A). Considering that the MW of albumin in Abx and BM(PEG)_2_ were 67,096 g/mol and 308.29 g/mol, respectively, the conjugation ratio of BM(PEG)_2_ was calculated to be 62%. Subsequently, the MW of IL4R-Abx was determined to be a mixture of 67,827 and 68,273 g/mol, indicating the presence of Abx-BM(PEG)_2_ and IL4R-Abx with one IL4RPep-1 conjugated. The modification of Abx with IL4RPep-1 preserved the physical properties of Abx, including its size of approximately 160 nm and a spherical structure through analysis using DLS (Figure 1C and S1B-C) and TEM (Figure 1D and S1D), respectively.

Additionally, using FITC-conjugated IL4RPep-1, the conjugation efficiency was determined by analyzing the absorbance of Abx and FITC at 280 and 495 nm, respectively, following the quantification method of dye-to-protein ratio. The extinction coefficient and correction factor were calculated from each absorbance spectra of Abx and FITC-IL4RPep-1 (Figure S2A-E). Subsequently, the conjugation efficiency was calculated from the absorbance of FITC-IL4R-Abx at 280 and 495 nm, applying constant values (Figure S2F and S2G). The resulting conjugation efficiency was calculated to be 59.69%, corresponding to approximately 0.6 molecules of IL4RPep-1 per molecule of albumin. These results indicated that IL4RPep-1 was successfully conjugated to Abx.

### IL4R-targeted Abx was internalized into M2 macrophages through IL4R-mediated endocytosis

**Figure 2:**
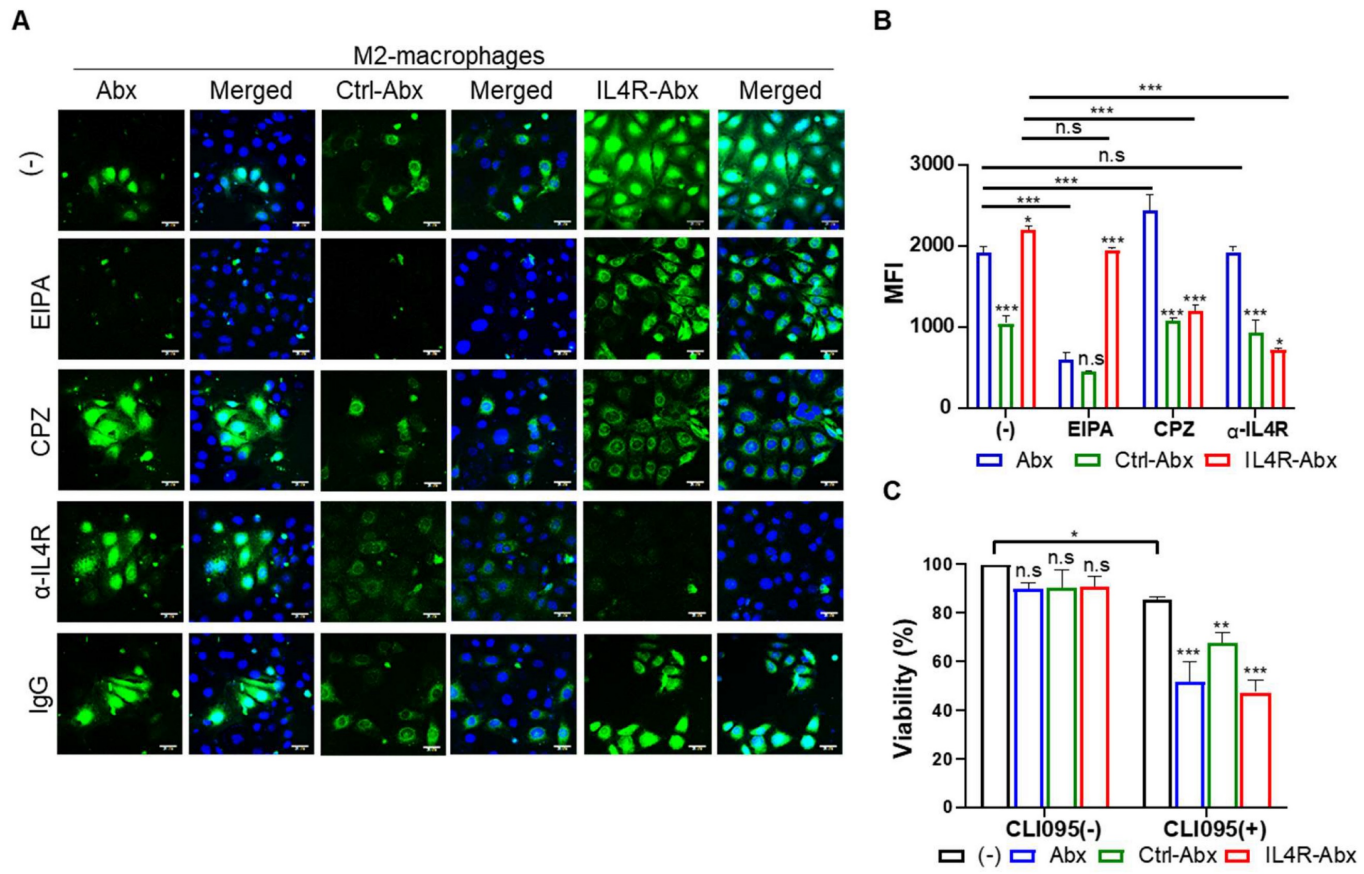
Internalization and cytotoxicity of IL4R-Abx in M2 macrophages. **(A)** Immunofluorescence analysis of the internalization. M2 macrophages were incubated using 2 μg/mL of fluorescein isothiocyanate (FITC) dye (green)-labeled Abx, Ctrl-Abx, and IL4R-Abx for 2 h with or without pre-treatment with 5-(n-ethyl-n-isopropyl) amiloride (EIPA) (50 μM), chlorpromazine (CPZ) (50 μM), anti-IL4R antibody (100 μM), and IgG (100 μM) for 30 min. Nucleus was stained with 4',6-diamidino-2-phenylindole (DAPI) (blue), and merging of images was performed. Scale bars, 20 μm. **(B)** Flow cytometry analysis of internalization. M2 macrophages were treated as described in (A), and the mean fluorescence intensity (MFI) was quantified using a flow cytometer. Data are expressed as mean ± standard deviation (SD) in the three separate experiments. *P* values of Ctrl-Abx and IL4R-Abx compared to Abx in each group are shown on the top of bars. *, *P* < 0.05; **, *P* < 0.01; ***, *P* < 0.001; n.s., not significant through one-way analysis of variance (ANOVA) followed by Tukey's multiple post hoc test. **(C)** Cytotoxicity assay. M2 macrophages were treated with 5 μg/mL of Abx, Ctrl-Abx, and IL4R-Abx for 24 h with or without pre-treatment with 500 nM of CLI095 for 30 min and subjected to cytotoxicity assays. Data are expressed as mean ± SD in the three separate experiments. *P* values compared to untreated control in each group are shown on the top of bars. *, *P* < 0.05; **, *P* < 0.01; ***, *P* < 0.001; n.s., not significant by two-way ANOVA followed by Bonferroni multiple post hoc test.

IL4R-Abx was internalized into M2 macrophages more efficiently than the unmodified Abx and control peptide-conjugated Abx (Ctrl-Abx) as determined by fluorescence microscopy (Figure 2A) and mean fluorescence intensity (MFI, Figure 2B) and percent internalization (Figure S3A and S3B) by flow cytometry. Ctrl-Abx was included to examine whether the conjugation of Abx with peptides affected the targeting activity of Abx regardless of the peptide sequence. Abx is known to be internalized into macrophages through macropinocytosis 8. Macropinocytosis is a receptor-independent and actin-driven endocytic pathway 32. To examine whether IL4R-Abx is internalized into macrophages via macropinocytosis, M2 macrophages were pre-treated with EIPA, a macropinocytosis inhibitor, before treatments with IL4R-Abx. EIPA efficiently inhibited Abx internalization while minimally reducing that of IL4R-Abx (Figure 2A-B and S3A-B). Conversely, pre-treatment with CPZ, a clathrin-mediated endocytosis inhibitor, efficiently reduced IL4R-Abx internalization while increasing that of Abx (Figure 2A-B and S3A-B). Cells were pre-treated with an anti-IL4R blocking antibody as the clathrin-mediated endocytosis is a receptor-dependent pathway. An anti-IL4R antibody significantly inhibited IL4R-Abx internalization while demonstrating a negligible effect on Abx (Figure 2A-B and S3A-B). Ctrl-Abx internalization was lower than Abx and IL4R-Abx and reduced by EIPA while being not inhibited by CPZ and anti-IL4R antibody (Figure 2A-B and S3A-B). In addition, EIPA and CPZ reduced the IL4R-Abx internalization but did not appear to hinder its cell surface binding, while the anti-IL4R antibody inhibited both the internalization and surface binding of IL4R-Abx (Figure 2A and 2B). These results indicate that IL4R-Abx internalization into M2 macrophages is a primarily IL4R targeted and the receptor-mediated endocytosis rather than macropinocytosis.

M2 macrophage treatments with Abx, Ctrl-Abx, and IL4R-Abx failed to significantly affect cell viability compared to untreated control (Figure 2C). However, M2 macrophage pre-treatment with CLI095, a TLR4 inhibitor, reduced cell viability through the above treatments compared to untreated control (Figure 2C), suggesting TLR4 involvement in the M2-macrophage viability against IL4R-targeted and untargeted Abx.

### IL4R-Abx enhanced M2-macrophage reprogramming into M1-like phenotype

**Figure 3:**
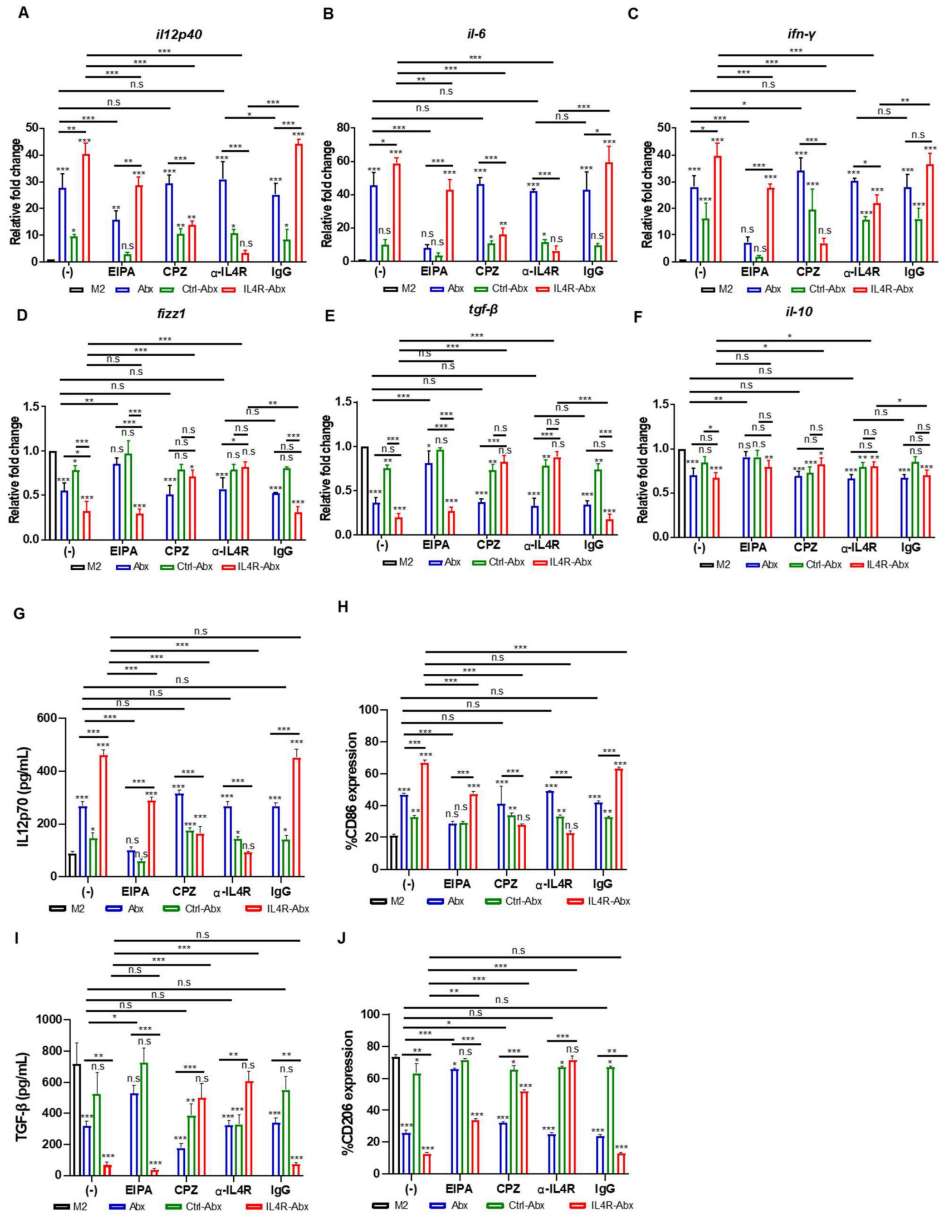
Reprogramming of M2 macrophages into M1-like phenotype by IL4R-Abx.** (A-F)** M2 macrophages were incubated with 5 μg/mL of Abx, Ctrl-Abx, and IL4R-Abx for 24 h without (-) or with pre-treatment with EIPA (50 μM), CPZ (50 μM), anti-IL4Rα antibody (100 μM), and IgG (100 μM) for 30 min. Relative mRNA levels of *il12p40* **(A)**, *il6* **(B)**, *ifn-γ* **(C)**, *fizz1* **(D)**, *tgf-β* **(E)**, and *il10* **(F)** were analyzed using quantitative reverse transcription-polymerase chain reaction (qRT-PCR) using a real time cycler. **(G, I)** ELISA of the concentrations of IL12p70 **(G)** and TGF-β **(I)** secreted into the culture medium. **(H, J)** Flow cytometry analysis of CD86 **(H)** and CD206 **(J)** expression. Data are expressed as mean ± SD in the three separate experiments.* P* values compared to untreated M2-macrophage control (M2, black bar) are shown on the top of bars. *, *P* < 0.05; **, *P* < 0.01; ***, *P* < 0.001; n.s., not significant by one-way ANOVA followed by Tukey's multiple post hoc test.

Abx and paclitaxel reprogram M2-type macrophages into the M1 phenotype 6, 8. M2 macrophages were treated with untargeted and IL-4R-targeted Abx to examine the reprogramming of the macrophage phenotype. qRT-PCR analysis revealed that the relative mRNA levels of M1-macrophage markers, such as *il12p40*,* il6*, and* ifn-γ*, were significantly increased after IL4R-Abx treatment compared with untreated control, Abx, and Ctrl-Abx (Figure 3A-C). Conversely, the relative mRNA levels of M2-macrophage markers, such as *fizz1*, *tgf-β,* and *il-10*, were significantly decreased after IL4R-Abx treatment compared to untreated control and Ctrl-Abx, while the decreases in those of *tgf-β* and *il-10* were not significant compared with Abx (Figure 3D-F). Compared to *fizz1* and *tgf-β*, the decreases in the relative mRNA levels of *il-10* by Abx and IL4R-Abx were weaker and showed lower responses (Figure 3F). In addition to the relative mRNA levels, the protein levels of M1-macrophage markers, such as IL12p70 (Figure 3G), CD86 (Figure 3H and S4A), and iNOS (Figure S4C), were increased after IL4R-Abx treatment compared with untreated control, Abx, and Ctrl-Abx. Conversely, the protein levels of M2-macrophage markers, such as TGF-β (Figure 3I), CD206 (Figure 3J and S4B), and Arg1 (Figure S4C), were decreased after IL4R-Abx treatment compared with untreated control, Abx, and Ctrl-Abx. Ctrl-Abx demonstrated weaker effects on the levels of M1- and M2-macrophage markers than unmodified Abx, indicating that Abx conjugation with non-targeting peptide interferes with naïve Abx activity (Figure 3A-J). These results suggest that IL4R-Abx reprograms the M2 macrophage into M1-like phenotype more efficiently than untargeted and control peptide-labeled Abx.

Pre-treatment of M2 macrophages with EIPA hampered the increase of M1-macrophage markers, including relative mRNA levels of *il12p40*,* il6*, and* ifn-γ* (Figure 3A-C) and protein levels of IL12p70 (Figure 3G) and CD86 (Figure 3H and S4A), by Abx and IL4R-Abx. EIPA failed to inhibit the decrease of M2-macrophage markers, including relative mRNA levels of *fizz1*, *tgf-β*, and* il-10* (Figure 3D-F), protein levels of TGF-β (Figure 3I), and CD206 (Figure 3J and S4B), by IL4R-Abx while still efficiently inhibiting that of Abx. Contrarily, M2-macrophage pre-treatment with CPZ and anti-IL4R blocking antibody remarkably inhibited the increase of M1-macrophage related mRNA and protein levels and the decrease of M2-macrophage related mRNA and protein levels by IL4R-Abx, while demonstrating negligible effects by Abx (Figure 3A-J and S4A-B).

### IL4R-Abx reprograms M2 macrophages to M1-like phenotype via the ROS-HMGB1-TLR4 axis

**Figure 4:**
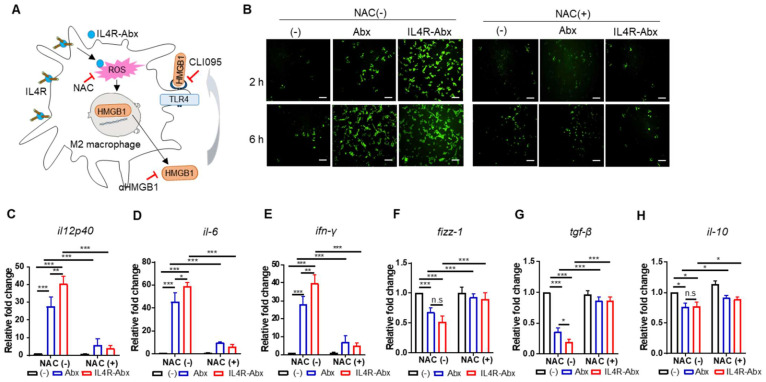
IL4R-Abx reprograms M2 macrophages into M1-like phenotype via increase in ROS. **(A)** Suggested pathways and inhibitors of IL4R-Abx-induced M2-macrophage reprogramming. **(B)** ROS analysis. M2 macrophages were incubated with 5 μg/mL of Abx and IL4R-Abx for 2 and 6 h with or without pre-treatment with 50 mM of N-acetyl-l-cysteine (NAC) for 30 min. A fluorescent microscope was used to image the fluorescence of H_2_DCFDA generated by ROS. **(C-H)** M2 macrophages were incubated with 5 μg/mL of Abx and IL4R-Abx for 24 h with or without pre-treatment with 50 mM of NAC for 30 min. Relative mRNA levels of M1-macrophage markers, such as *il12p40* **(C)**, *il-6* **(D)**, and *ifn-γ* **(E)**, and M2-macrophage markers, such as *fizz-1* **(F)**, *tgf-β* **(G)**, and *il-10* **(H)**, were measured via qRT-PCR analysis. (-), untreated M2-macrophage control (black bar).

Paclitaxel increases ROS accumulation and triggers the extracellular release of HMGB1 from macrophages, which contributes to paclitaxel-induced peripheral neuropathy 33. Extracellular HMGB1 binds to TLR4 and activates pro-inflammatory cytokine secretion and skews M2 macrophage to the M1 phenotype 34-37. According to these previous findings, we explored whether Abx and IL4R-Abx shape M2 macrophages toward the M1 phenotype via ROS-HMGB1-TLR4 axis stimulation (Figure 4A). IL4R-Abx increased the ROS levels in M2 macrophages at higher levels at 2 h after treatment compared to untreated cells and Abx, while the increases in ROS levels by Abx and IL4R-Abx were similar at 6 h after treatments, which were blocked by pre-treatment of cells with NAC, an ROS scavenger (Figure 4B). Moreover, NAC significantly impeded the increase of the relative mRNA levels of M1-macrophage markers (*il12p40, il-6*, and *ifn-γ*; Figure 4C-E), including the decrease of M2-macrophage markers (*fizz-1, tgf-β*, and *il-10*; Figure 4F-H) by Abx and IL4R-Abx. These results indicate that IL4R-Abx more rapidly and efficiently increases ROS levels in M2 macrophages and induces the skew of the macrophage polarity compared to Abx.

**Figure 5:**
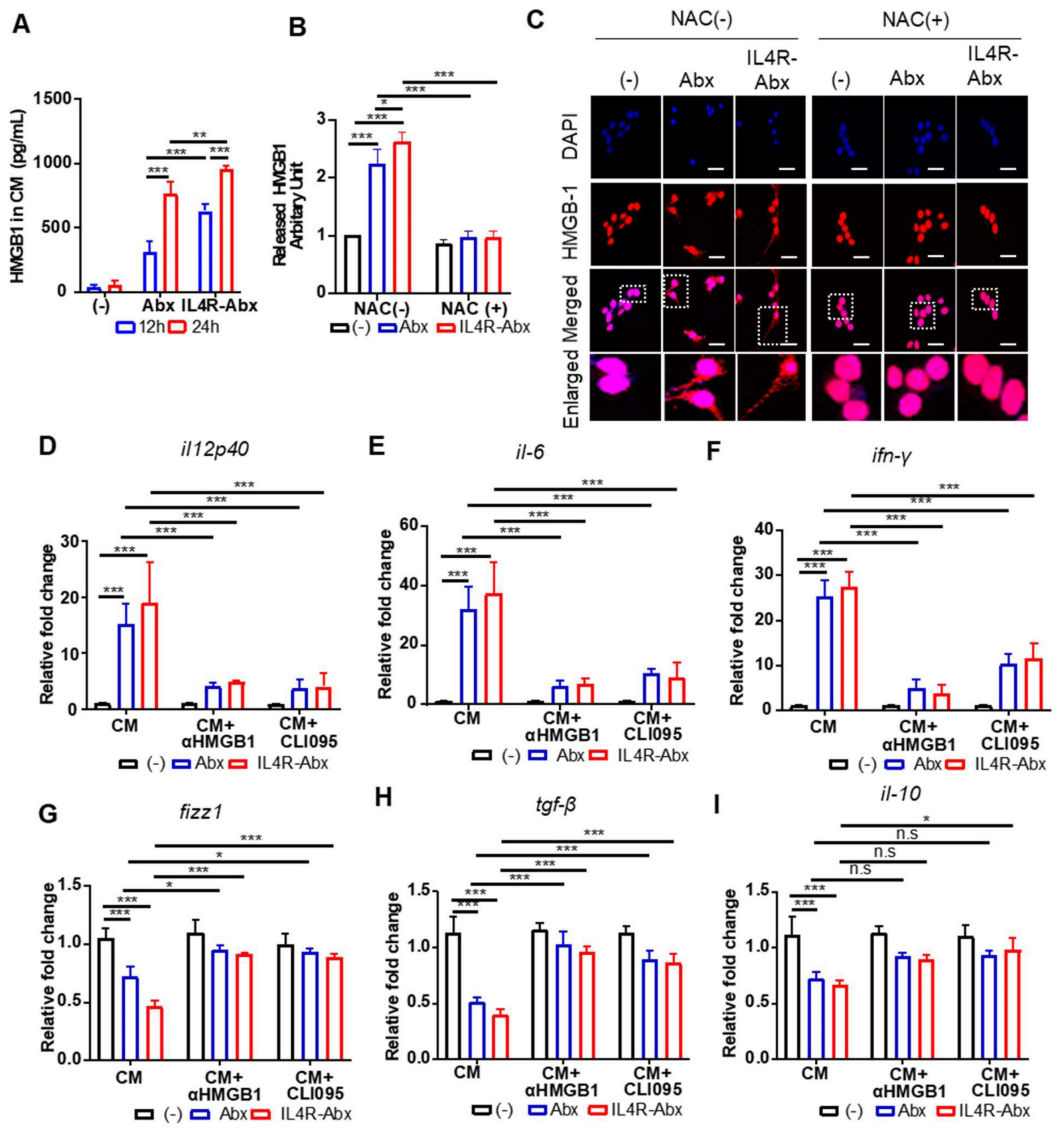
IL4R-Abx reprograms M2 macrophages into M1-like phenotype via the ROS-HMGB1-TLR4 axis. **(A)** M2 macrophages were incubated with 5 μg/mL of Abx and IL4R-Abx for 12 and 24 h. Concentrations of HMGB1 released into the conditioned medium were determined by ELISA. **(B)** M2 macrophages were incubated with 5 μg/mL of Abx and IL4R-Abx for 24 h with or without pre-treatment with 50 mM of NAC for 30 min. The conditioned medium was collected, concentrated, and subjected to western blotting using an anti-HMGB1 antibody. HMGB1 protein band intensity was quantified using Image J software. **(C)** M2 macrophages were incubated with Abx and IL4R-Abx for 24 h with or without pre-treatment with NAC. Cells were incubated with anti-HMGB1 antibody (red). Nucleus was stained with DAPI (blue), and images were merged. Boxes represent the enlarged area. Scale bars, 40 µm.** (D-I)** M2 macrophages were incubated using Abx or IL4R-Abx for 12 h with or without pre-treatment of anti-HMGB1 antibody (10 μg/mL, CM+αHMGB1) and CLI095 (500 nM, CM+CLI095) for 30 min. The CM of M2 macrophages was collected and treated to other sets of M2 macrophages for 24 h. Relative mRNA levels of M1-macrophage markers, such as *il12p40* **(D)**, *il-6* **(E)**, and *ifn-γ* **(F)**, and M2-macrophage markers, such as *fizz-1* **(G)**, *tgf-β* **(H)**, and *il-10* **(I)**, were measured via qRT-PCR analysis. Data are expressed as mean ± SD in the three separate experiments. *, *P* < 0.05; **, *P* < 0.01; ***, *P* < 0.001; n.s., not significant by one-way ANOVA followed by Tukey's multiple post hoc test. (-), untreated M2-macrophage control (black bar).

Next, the extracellular release and nuclear to cytoplasmic translocation of HMGB1 and subsequent activation of TLR4 after treatments with Abx and IR4R-Abx were examined. IL4R-Abx increased the extracellular release of HMGB1 into the CM of M2 macrophages as determined by ELISA (Figure 5A) and western blotting (Figure 5B and S5) more efficiently than Abx. In the same context, the nuclear to cytoplasmic translocation of HMGB1 was more remarkable after treatment with IL4R-Abx compared to Abx (Figure 5C). The extracellular release and nuclear to cytoplasmic translocation of HMGB1 were blocked by pre-treatment with NAC (Figure 5B, 5C, and S5). The CM of M2 macrophages treated with IL4R-Abx increased the relative mRNA levels of M1-macrophage markers (Figure 5D-F) and decreased those of the M2-macrophage markers (Figure 5G-I) in recipient M2 macrophages cultured on separate plates more efficiently than the CM of M2 macrophages incubated with Abx. Pre-treatment of recipient M2 macrophages with an anti-HMGB1 blocking antibody inhibited the alterations in the macrophage polarity markers induced by CM treatment (Figure 5D-I). Moreover, pre-treatment of recipient M2 macrophages with CLI095 also impeded the changes in the macrophage polarity markers induced by CM treatment (Figure 5D-I). These results indicate that after being internalized into M2 macrophages, Abx and IL4R-Abx skew them into M1-like phenotype through the ROS-HMGB1-TLR4 axis.

### IL4R-Abx selectively homes to tumors in mice

**Figure 6:**
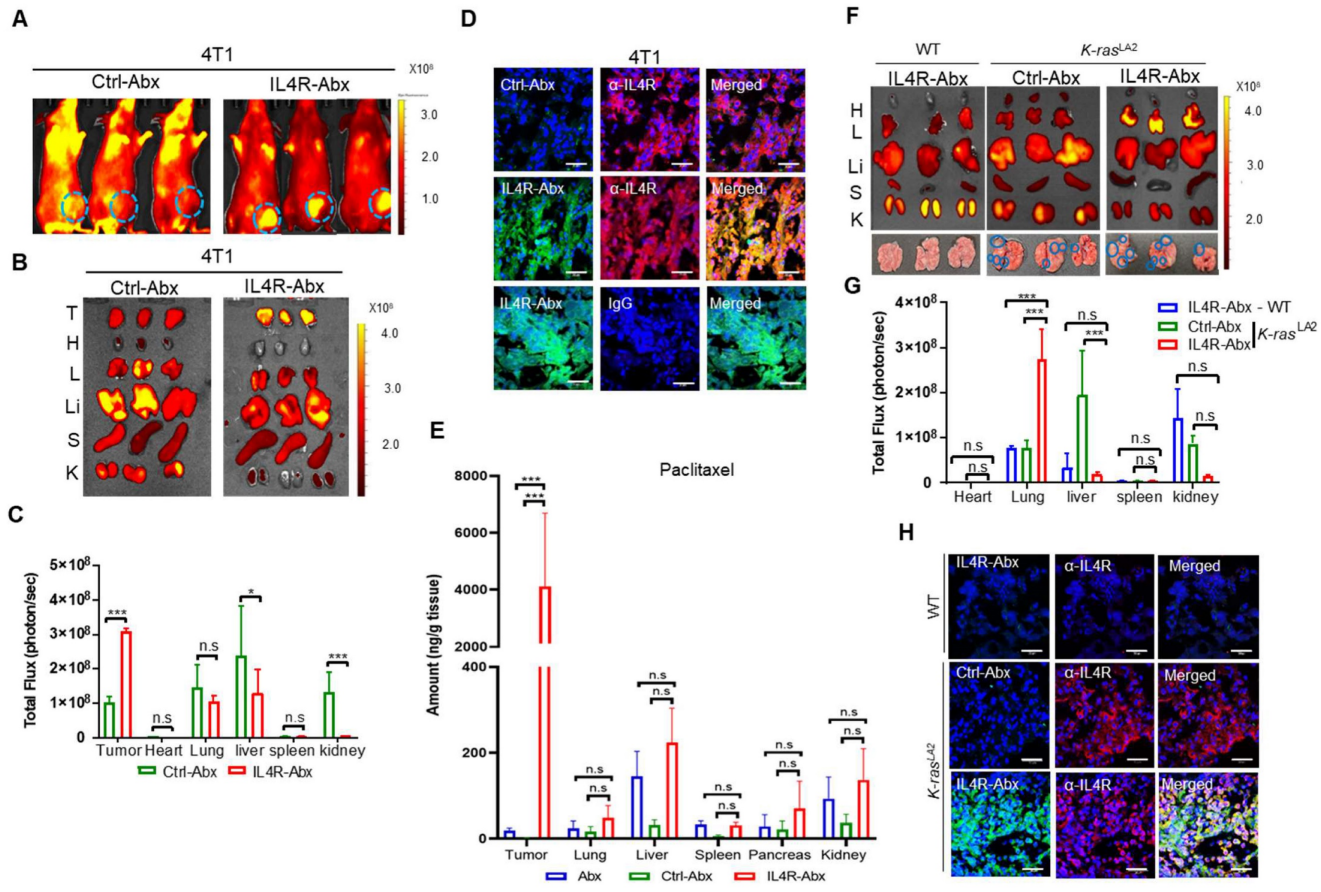
Tumor homing of IL4R-Abx in mice bearing 4T1 breast tumor and *K-ras^LA2^* lung tumor. **(A)** Whole-body fluorescence imaging of mice bearing 4T1 tumor at 6 h after injection of near-infrared fluorescence dye-labeled Ctrl-Abx and IL4R-Abx (5 mg/kg body weight). Circles in cyan blue represent the region of interest (tumor site). **(B, C)** *Ex vivo* imaging of tumor and organs isolated after whole-body imaging **(B)** and total flux quantification (photon/s) of each organ **(C)**. T, tumor; H, heart; L, lung; Li, liver; S, spleen; K, kidney. **(D)** Histochemical analysis. Tumor tissues isolated at 6 h after injection of FITC dye (green)-labeled Ctrl-Abx and IL4R-Abx underwent frozen section. Tissues were incubated using an anti-IL4R antibody or IgG (red) and with DAPI for nucleus staining (blue), and images were merged. Scale bars, 20 μm. **(E)** Amount of paclitaxel (ng/g tissue) in tumor and control organs were determined at 6 h after Abx, Ctrl-Abx, and IL4R-Abx injection (5 mg/kg body weight) in mice bearing 4T1 tumor. **(F, G)** *Ex vivo* imaging of tumor and organs isolated at 6 h after Ctrl-Abx and IL4R-Abx injection (5 mg/kg body weight) into *K-ras^LA2^* mutant and wild-type (WT) littermate mice **(F)** and quantification of the total flux (photon/sec) of each organ **(G)**. H, heart; L, lung; Li, liver; S, spleen; K, kidney. Circles (cyan blue) reflect tumor nodules in the lungs. **(H)** Histochemical analysis. Tumor tissues isolated at 6 h after injection of FITC dye (green)-labeled Ctrl-Abx and IL4R-Abx into *K-ras^LA2^* mutant and WT littermate mice and then were subjected to frozen section. An anti-IL4R antibody (red) was used for tissue staining, with DAPI for nucleus staining (blue), and images were merged. Scale bars, 20 μm. Data are expressed as mean ± SD *, *P* < 0.05; ***, *P* < 0.001; n.s., not significant by two-way ANOVA followed by Bonferroni post hoc test (*n* = 3 per group).

For *in vivo* fluorescence imaging, IL4R-Abx and Ctrl-Abx were conjugated with near-infrared fluorescence dye-labeled IL4RPep-1 and control peptide, respectively, and injected into mice bearing 4T1 breast tumor at the mammary fat pad via the tail vein. Whole-body fluorescence imaging acquired at 6 h after IL4R-Abx injection revealed higher levels of fluorescence signals at the tumor site and lower levels of background signals compared to Ctrl-Abx (Figure 6A). *Ex vivo* imaging and fluorescence intensity quantification of tumors and control organs isolated after the *in vivo* imaging also demonstrated higher levels of uptake at tumors and lower levels of accumulation at control organs, particularly at the liver, by IL4R-Abx than Ctrl-Abx (Figure 6B and 6C). Histological analysis further revealed higher levels of accumulation by IL4R-Abx than Ctrl-Abx in 4T1 tumor tissues where IL4R was abundantly expressed (Figure 6D). To examine paclitaxel amounts accumulated at tissues, mice bearing 4T1 breast tumor at the mammary fat pad were injected via the tail vein with targeted and untargeted Abx. Tumor and control organs such as the lung and liver were isolated, and the amounts of paclitaxel in tissues (ng/g tissue) were determined through mass spectrometry. In tumor tissues, significantly higher amounts of paclitaxel were detected with minimal accumulation at control organs in mice injected with IL4R-Abx compared to Abx and Ctrl-Abx (Figure 6E).

In addition to 4T1 breast tumor, we investigated tumor homing of IL4R-Abx in two lung tumor models: *K-ras^LA2^* mutant transgenic mouse tumor and LLC syngeneic mouse tumor. *Ex vivo* imaging of lungs and control organs at 6 h after injection demonstrated that IL4R-Abx accumulated at lungs of *K-ras^LA2^* mutant mice bearing tumor nodules at higher levels than at control organs such as the liver, whereas Ctrl-Abx accumulated mostly in the liver (Figure 6F and 6G). IL4R-Abx accumulation at lungs of WT mice was as low as Ctrl-Abx (Figure 6F and 6G). Histological analysis revealed higher levels of IL4R-Abx accumulation and IL4R expression in the lung tumor nodules of *K-ras^LA2^* mutant mice compared with lung tissues of WT mice (Figure 6H). A similar pattern of tumor homing by IL4R-Abx was noted in mice bearing subcutaneous LLC lung tumor (Figure S6A-C). Moreover, higher levels of tumor accumulation of IL4R-Abx compared to Ctrl-Abx were observed up to 24 h after injection in Panc1 subcutaneous human pancreatic xenograft tumor (Figure S7A-D). These results suggested that IL4R-Abx more selectively homed to IL4R-high tumors with minimal accumulation at control organs than Abx and Ctrl-Abx.

### IL4R-Abx inhibits tumor growth and metastasis and improves antitumor immunity

**Figure 7:**
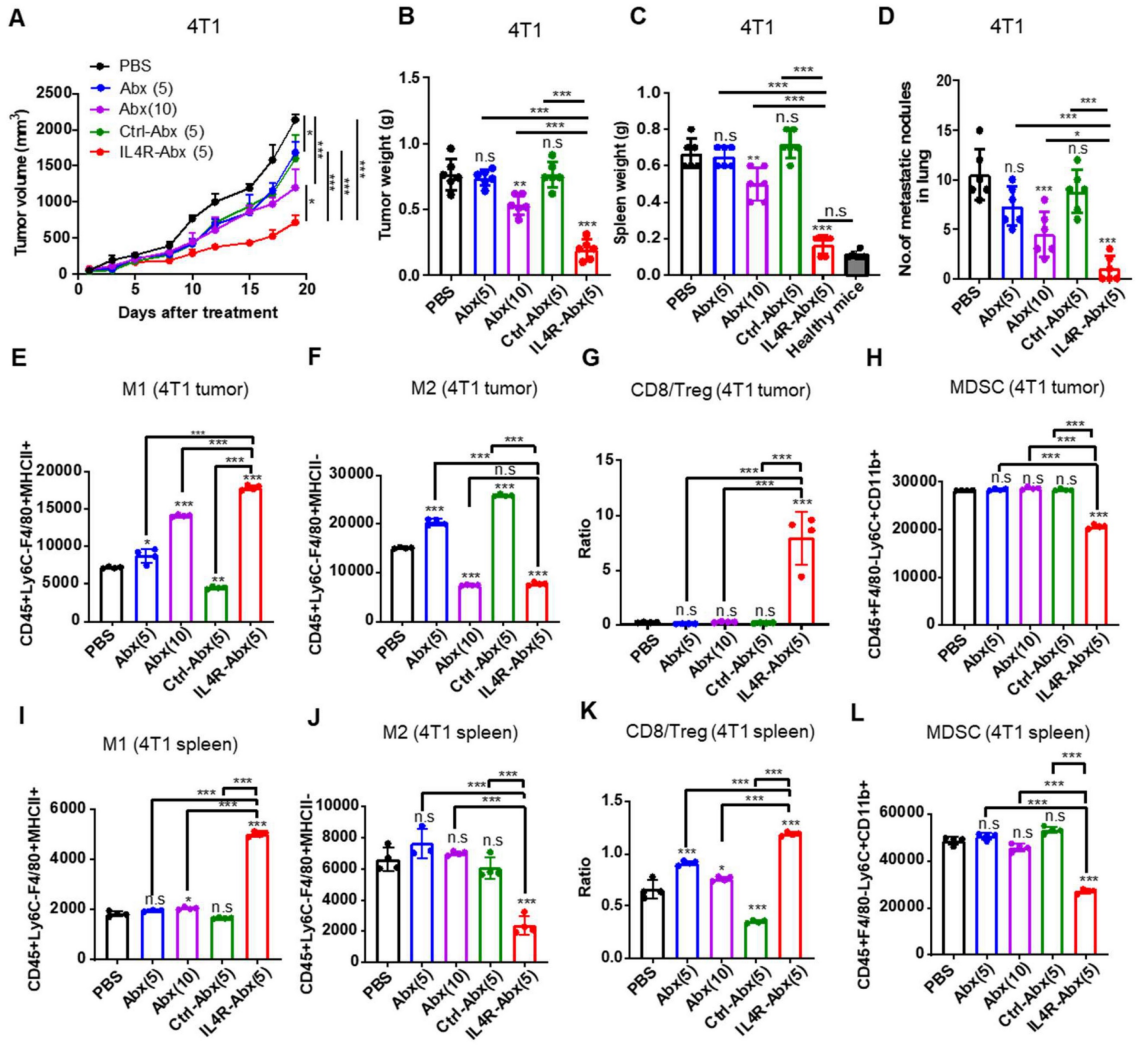
Inhibition of tumor growth and metastasis and improvement of antitumor immunity by IL4R-Abx in mice bearing 4T1 breast tumor. **(A-D)** Mice bearing 4T1 tumor were injected with Abx, Ctrl-Abx, and IL4R-Abx (5 or 10 mg/kg body weight, weekly for 4 weeks). The tumor volume **(A)**, tumor weight **(B)**, spleen weight **(C)**, and number of metastatic nodules in the lungs **(D)** after treatments were measured. **(E-L)** At the culmination of the treatments, tumor and spleen were isolated for immune cell analysis using flow cytometry. The population of CD45+F4/80+Ly6C-MHC II+ M1 macrophages **(E)**, CD45+F4/80+Ly6C-MHC II
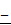
M2 macrophages **(F)**, CD8/Treg ratio **(G)**, and CD45+F4/80-Ly6C+CD11b+ MDSCs **(H)** in 0.1 g of tumor tissues and the population of M1 macrophages **(I)**, M2 macrophages **(J)**, CD8/Treg ratio **(K)**, and MDSCs **(L)** in 10^5^ cells of spleen were determined. Data are expressed as mean ± SD. *P* values compared to saline-treated group are shown on the top of bars. *, *P* < 0.05; **, *P* < 0.01; ***, *P* < 0.001; n.s., not significant by one-way ANOVA followed by Tukey's multiple post hoc test. (A, *n* = 10 per group; B-D, *n* = 6 per group; E-L, *n* = 4 per group).

Mice bearing 4T1 tumor were injected intravenously (weekly for 4 weeks) with IL4R-targeted and untargeted Abx. IL4R-Abx decreased the tumor volumes (Figure 7A), tumor weights (Figure 7B), spleen weights or splenomegaly (Figure 7C and S8A), and the number of metastatic tumor nodules in the lungs (Figure 7D and S8B) more efficiently than Abx and Ctrl-Abx given at a similar dose (5 mg/kg body weight) and Abx given at a higher dose (10 mg/kg body weight). The decrease in the number of lung metastatic tumor nodules through Abx treatment was attained only at a 10 mg/kg body weight dose (Figure 7D). Next, immune cell population in tumor tissue and spleen were analyzed after treatments using flow cytometry (Figure S9A). Compared to Abx and Ctrl-Abx, IL4R-Abx more efficiently increased the population of M1-macrophage (Figure 7E and S9B) and CD8+ T-cells (Figure S9B and S9C) and CD8+ T-cell/Treg ratio (Figure 7G), while reducing the population of M2 macrophages (Figure 7F and S9B), MDSCs (Figure 7H and S9B), and Tregs (Figure S9B and S9D) in the tumor microenvironment. Conversely, untargeted Abx did not significantly change CD8+ T-cell/Treg ratio (Figure 7G) and the population of MDSC, CD8+ T-cells, and Tregs in tumor tissues (Figure 7H and S9B-D) even at a higher dose (10 mg/kg body weight). Similar to tumor tissues, IL4R-Abx changed immune cell population in the spleen of tumor-bearing mice and improved antitumor immunity more efficiently than Abx and Ctrl-Abx (Figure 7I-L).

**Figure 8:**
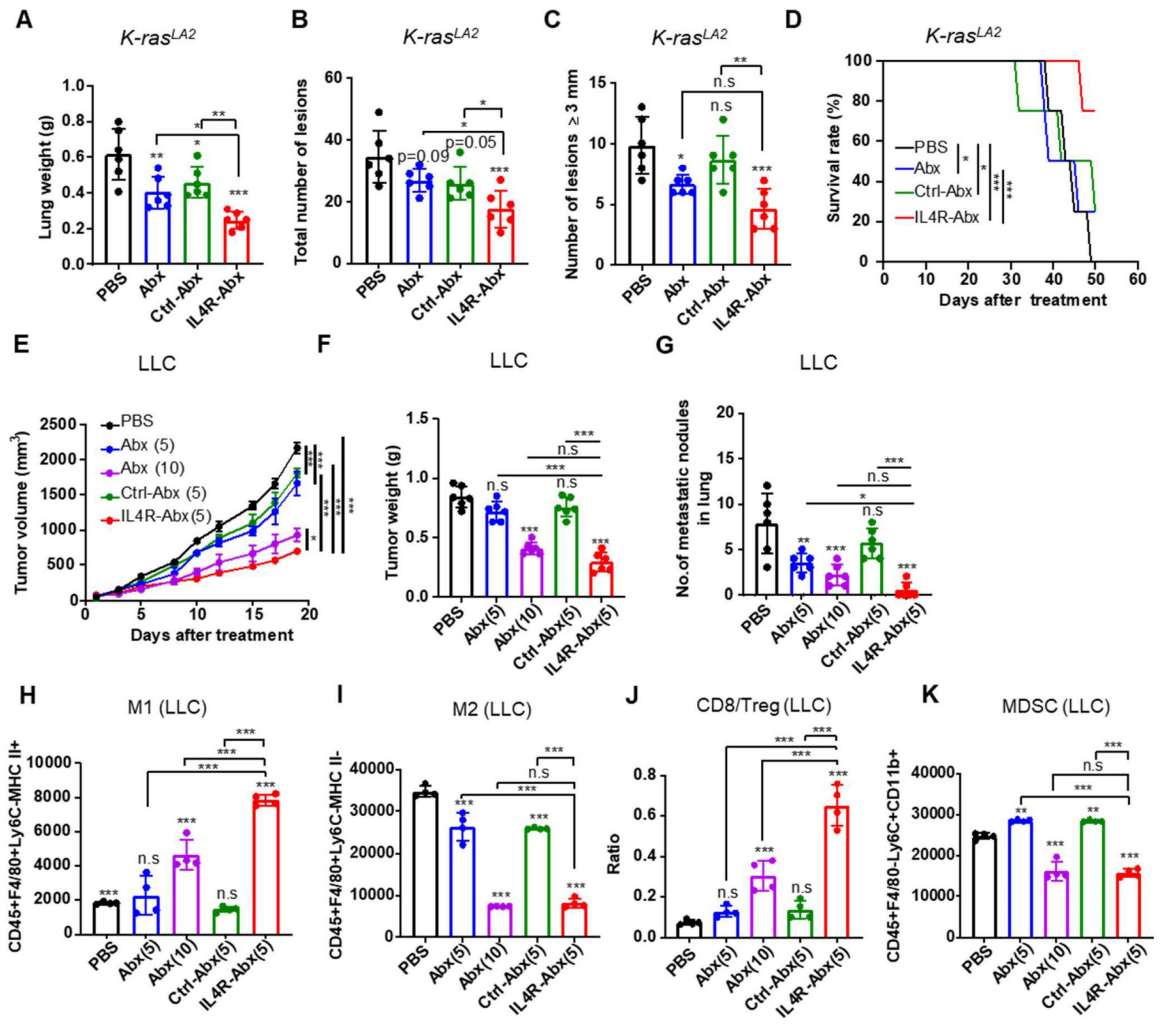
Therapeutic efficacy of IL4R-Abx in *K-ras^LA2^* mutant and LLC lung tumor-bearing mice.** (A-D)** *K-ras^LA2^* mutant mice bearing spontaneous lung tumors were intravenously injected with Abx, Ctrl-Abx, and IL4R-Abx (5 mg/kg body weight, weekly for 4 weeks). The lung weight** (A)**, the total number of tumor lesions in the lungs **(B)**, the number of tumor lesions above 3 mm in diameter **(C)**, and survival rate **(D)** at the culmination of the treatments were determined. **(E-K)** Mice bearing subcutaneous LLC lung tumor were injected intravenously with Abx, Ctrl-Abx, and IL4R-Abx (5 or 10 mg/kg body weight, weekly for 4 weeks). The tumor volume** (E)**, tumor weight **(F)**, and the number of metastatic nodules in the lungs **(G)** were determined. The population of CD45+F4/80+Ly6C-MHC II+ M1 macrophages **(H)**, CD45+F4/80+Ly6C-MHC II-M2 macrophages **(I)**, CD8/Treg ratio **(J)**, and CD45+F4/80-Ly6C+CD11b+ MDSCs **(K)** in 0.1 g of tumor tissues were determined via flow cytometry. Data are expressed as mean ± SD. *P* values compared to the saline-treated group are shown on the top of bars. *, *P* < 0.05; **, *P* < 0.01; ***, *P* < 0.001; n.s., not significant by one-way ANOVA followed by Tukey's multiple post hoc test. (A-D, *n* = 6 per group; E, *n* = 10 per group; F-G, *n* = 6 per group; H-K, *n* = 4 per group).

Next, the therapeutic efficacy of IL4R-Abx in mouse lung tumor models was investigated. Ten-week old* K-ras^LA2^* mutant mice and mice bearing LLC lung tumors were injected with IL4R-targeted and untargeted Abx (5 or 10 mg/kg body weight, weekly for 4 weeks). IL4R-Abx reduced the lung weights (Figure 8A), the total number of tumor lesions (Figure 8B and S10), and the number of tumor lesions above 3 mm in diameter (Figure 8C) and increased survival rates (Figure 8D) in *K-ras^LA2^* mutant mice more efficiently than Abx and Ctrl-Abx, while no statistical difference was observed for the number of large lesions (above 3 mm in diameter) compared to Abx. In LLC lung tumor mice, IL4R-Abx decreased the tumor volumes (Figure 8E), tumor weights (Figure 8F), and number of metastatic tumor nodules in the lungs (Figure 8G) more efficiently than Abx and Ctrl-Abx. Additionally, IL4R-Abx increased the M1-macrophage population (Figure 8H) and CD8+ T-cell/Treg ratio (Figure 8J), while decreasing the M2-macrophage (Figure 8I) and MDSC population (Figure 8K) in LLC tumor tissues at higher levels than Abx and Ctrl-Abx. The reduction in the tumor volumes and increases in the M1-macrophage population and CD8/Treg ratio by IL4R-Abx were higher than those by Abx given at a higher dose (10 mg/kg body weight), while the decline in tumor weights, the number of metastatic nodules, and the M2-macrophage and MDSC population by IL4R-Abx was equivalent to those of Abx given at a higher dose (Figure 8E-K).

We used two pancreatic tumor models to further examine the antitumor effects of IL4R-Abx: KPC transgenic mice and Panc-1 human pancreatic tumor xenografts. KPC mice spontaneously developed pancreatic intraepithelial neoplasia at 8-10 weeks of age and invasive pancreatic ductal adenocarcinoma by the 16th week 38. IL4R expression increased during 9-18 weeks of age in KPC mice (Figure S11A). IL4R-Abx decreased the population of Ki67+ proliferating cells and IL4R-expressing cells in tumor tissues more efficiently than Abx when systemically administered at 10 weeks of age (5 mg/kg body weight, weekly for 4 weeks) (Figure S11B). In the pancreatic tumor tissues of KPC mice, IL4R-Abx increased the M1-macrophage population (Figure S11C) and decreased the MDSC population (Figure S11F) at higher levels than Abx, while the M2-macrophage population (Figure S11D) and CD8+ T-cell/Treg ratio (Figure S11E) were equivalent to those of Abx. In Panc-1 human pancreatic tumor xenografts, IL4R-Abx inhibited the tumor volumes (Figure S12A), tumor weights (Figure S12B), number of metastatic nodules in the lungs (Figure S12C), and TUNEL+ apoptosis levels (Figure S12D) more effectively than Abx.

Serum levels of liver enzymes, such as AST and ALT, and kidney function indicators, such as BUN and creatinine, were measured at the culmination of the treatments to explore the systemic side effects of IL4R-Abx. IL4R-Abx did not induce a noticeable toxicity to the liver compared to saline; however, Abx increased the serum levels of AST in 4T1 (Figure S13A-D) and AST and ALT in *K-ras^LA2^* mutant mice (Figure S13E-H) as well as Panc-1 tumor xenograft-bearing mice (Figure S13I-L), suggesting liver toxicity. The serum levels of BUN and creatinine after Abx and IL4R-Abx treatments were within normal ranges, suggesting no kidney toxicity. In addition, analysis of hematological parameters showed that white blood cell and neutrophil counts were above normal ranges (Figure S14A and S14B), while lymphocyte, monocyte, red blood cell, and platelet counts were close to or within normal ranges (Figure S14C-F), in 4T1 tumor-bearing mice treated with Abx and IL4R-Abx as well as saline. Furthermore, hemolysis tests showed that IL4R-Abx as well as Abx, Ctrl-Abx, and IL4RPep-1 peptide did not induce hemolysis (Figure S15A and S15B), indicating the biocompatibility of IL4R-Abx in the blood.

While the IL4R-Abx induced 4T1, LLC, and Panc-1 tumor cell cytotoxicity at higher levels than Ctrl-Abx, it revealed similar cytotoxicity levels and IC50 values in tumor cells compared to Abx (Figure S16A-C). IL4R-Abx induced lower levels of cytotoxicity in HEK293 normal cells than Abx and Ctrl-Abx (Figure S16D), suggesting preferential Abx delivery to tumor cells over normal cells. These results indicated that a more efficient inhibition of tumor growth and metastasis by IL4R-targeted Abx over untargeted Abx was primarily attributed to its effects on the M2-macrophage polarity rather than tumor cell viability.

## Discussion

The study findings demonstrated that IL4R targeting allowed Abx to improve M2-macrophage reprogramming into an M1-like phenotype through the ROS-HMGB1-TLR4 axis and enhance the antitumor immunity via increasing immune-stimulatory cells while decreasing immune-suppressive cells in the tumor microenvironment, contributing to the inhibition of tumor growth and metastasis in tumor-bearing mouse models (Figure S17). Treatment with IL4R-Abx at a lower dose (5 mg/kg body weight) exerted higher levels of therapeutic efficacy compared to Abx (10 mg/kg body weight), suggesting IL4R targeting can reduce the dose of Abx. Moreover, IL4R-Abx enhanced survival rates and was accompanied by negligible systemic side effects such as liver toxicity compared to Abx*.* The IL4R-targeting activity was attained through the conjugation of the IL4R-targeting IL4RPep-1 peptide to a sulfur group exposed on the albumin surface of Abx. Abx has a core-shell structure with paclitaxel comprising the core through hydrophobic interactions, while albumin coats the paclitaxel, forming a stabilizing layer around the payload 39, 40. Given the layered nature of albumin, the conjugation reaction with albumin molecules at inner layers seems to be hindered by steric hindrance, resulting in the conjugation ratio of 60% in this study.

Compared to Abx, IL4R-Abx was more efficiently internalized into the M2 macrophages. The internalization of IL4R-Abx was primarily via the receptor-mediated endocytosis and was inhibited by an anti-IL4R antibody and a CPZ endocytosis inhibitor. Conversely, Abx was internalized primarily through the macropinocytosis as previously known 8. The increase in Abx internalization by CPZ observed in this study might be explained by previous findings that the inhibition of the clathrin-mediated endocytosis can increase the clathrin-independent endocytosis including macropinocytosis 41, 42. After internalization into macrophages, IL4R-Abx and Abx induced M2-macrophage reprogramming into M1-like phenotype through the ROS-HMGB1-TLR4 axis. IL4R-Abx and Abx increased intracellular ROS levels in M2 macrophages and extracellular release of HMGB1. NAC, a well-known ROS scavenger, inhibited intracellular ROS elevation and extracellular release of HMGB1 and subsequently inhibited the increase of M1-type cytokines and the decrease of M2-type cytokines induced by IL4R-Abx and Abx. Moreover, the CM of M2 macrophages pre-treated with IL4R-Abx and Abx elevated the M1-type cytokines and decreased the M2-type cytokines, indicating that an extracellular factor(s) is involved in this event. In the same context, paclitaxel has been shown to induce the extracellular release of HMGB1 in macrophages via the ROS/p38MAP kinase/NF-κB/histone acetyltransferase pathway 33. Furthermore, either an anti-HMGB1 antibody or a TLR4 inhibitor CLI095 efficiently hampered the alterations in the cytokine levels induced by the CM of M2 macrophages treated with Abx and IL4R-Abx. Collectively, our results indicate that IL4R-Abx and Abx enhanced ROS level upregulation, and the release of HMGB1 and extracellular HMGB1 bound to TLR4 and activated TLR4, resulting in pro-inflammatory activation of M2 macrophages. However, macrophages have an increased p-glycoprotein expression as an efflux pump of paclitaxel 43. Paclitaxel activates macrophages via a direct binding to TLR4 on the macrophage cell surface 6. These previous findings suggest that paclitaxel may be released from M2 macrophages treated with IL4R-Abx and Abx into the CM and contribute to the CM-induced TLR4 activation in collaboration with HMGB1. IL4R-targeted Abx and the unmodified Abx did not trigger cytotoxicity in the M2 macrophages, which corroborates with previous findings 6, 8, 43, and rather induced M2-macrophage repolarization. TLR4-mediated NF-κB activation promotes a resistance to Abx-mediated cell death 44, supporting the increase in cell death after Abx and IL4R-Abx treatments when TLR4 was inhibited by CLI095.

Whole-body imaging, histologic analysis, and measurement of paclitaxel concentrations in tissues showed that IL4R targeting allowed Abx to selectively accumulate at tumors avoiding control organs and thereby delivering more paclitaxel to tumors. *In vivo* and *ex vivo* fluorescence signals by the homing of nanoparticles tend to be dependent on the size and depth in the body of each organ. To accurately determine the amount of paclitaxel accumulated at organs by IL4R-Abx, the mass spectrometry quantification of paclitaxel in tissues was included. Organs such as the liver were homogenized, and the total amount of paclitaxel in the organ was measured and represented as an amount per tissue weight (ng/g tissue). Because the liver was larger than tumor, the relative amount of paclitaxel in the liver after IL4R-Abx treatment was lower compared to tumor, while the *ex vivo* fluorescence signals of the liver were apparently high. This may explain the discrepancy between the fluorescence images and paclitaxel amounts in the liver and tumor.

Targeted nanoparticle delivery, such as Abx, to tumors depends on the enhanced permeability and retention effect of nanoparticles through leaky tumor blood vessels while avoiding mature and tightly sealed blood vessels of normal tissues 45. An additional merit of albumin-bound nanoparticle formulation of paclitaxel is that it can eliminate the requirement for Cremophor, a toxic agent for solubility, and increase the circulation time of paclitaxel 45. Affinity-based targeting or targeted delivery of Abx via albumin modification with a tumor-homing peptide has been first exploited by Ruoslahti et al. 46. They conjugated Abx using CREKA peptide that binds to clotted plasma proteins in tumor blood vessels or LyP-1 peptide (CGNKRTRGC) that binds to p32 overexpressed on tumor cells; the CREKA- or LyP-1-guided Abx led to a significant inhibition of tumor growth compared to untargeted Abx 46. Furthermore, the combined treatment of Abx and CEND-1 (or iRGD) peptide, a cyclic peptide that targets alpha V-integrin and neurophilin-1 and improves internalization of co-administered drugs into tumor cells, demonstrated an increased activity and acceptable safety profile in a clinical trial 47. These results indicate that the direct Abx modification with a tumor-homing peptide or combined treatment of Abx and the iRGD internalizing peptide is a promising tool to improve tumor cell cytotoxicity of Abx.

Cancer therapy targeting M2 macrophages has been exploited in numerous methods 48, 49. For example, TLR3 and TLR7 agonists activated NF-κB and triggered secretion of immune-stimulatory cytokines in macrophages and have been in clinical trials for cancer therapy 49. An antibody against colony-stimulating factor-1 receptor hindered macrophage recruitment into tumors 50. An antibody against MARCO, a pattern recognition receptor, reprogrammed M2 macrophages into pro-inflammatory M1 macrophages 51. An agonistic anti-CD40 antibody triggered nitric oxide production in macrophages and inhibited tumor growth 52. In addition to antibodies, trabectedin, a chemotherapeutic drug, exerted antitumor growth activity via monocyte and macrophage cytotoxicity 53. Epigenetic therapies using DNA methylation and histone deacetylation inhibitors skewed M2 macrophages into the M1-like phenotype 54 and stimulated MDSC differentiation into a more-interstitial macrophage-like phenotype 55, leading to tumor progression and metastasis inhibition. Furthermore, M1-macrophage-derived exosomes engineered to target IL4R and stimulate pro-inflammatory polarization of macrophages skewed M2 macrophages toward M1-like macrophages 56.

Abx promotes multiple steps in the cancer-immunity cycle: antigen presentation; T-cell activation and infiltration; and tumor cell killing. Antigen peptides from tumor cells are generally presented to CD4+ T cells via MHC II on dendritic cells (DCs). Paclitaxel induces the expression of MHC II on DCs 57 and upregulates the expression of antigen processing machinery and costimulatory molecules such as CD86 in DCs 58. In addition, Abx promotes activation of CD8+ T cells by inhibiting the function of immune-suppressive cells including MDSCs and Tregs 59. Abx inhibits cancer-associated fibroblast proliferation and extracellular matrix synthesis, which promotes T-cell infiltration into tumor tissues 59. Moreover, Abx inhibits microtubule of tumor cells and coopts with CD8+ T cells to kill tumor cells. To our interest, MDSCs in the tumor microenvironment also express high levels of IL4R, and the IL4R expression is critical for their immune-suppressive activity on CD8+ T cells 60. These findings suggest that IL4R-Abx may enable an increased delivery of nab-paclitaxel to MDSCs as well as M2-macrophages, leading to more efficient inhibition of immune-suppressive myeloid cells compared to Abx. In addition to breast, lung, and pancreatic tumors exploited in this study, IL4R is upregulated in other types of tumors such as glioblastoma 61 and Hodgkin lymphoma 62. Collectively, these findings suggest that IL4R-targeted Abx has a broad spectrum of applications in the management of patients with cancer.

## Conclusions

Altogether, the current study suggests that IL4R-targeted Abx presents as a promising cancer immunotherapeutic agent targeting M2 macrophages.

## Supplementary Material

Supplementary methods and figures.
