# Supplementary material for: IL4 receptor targeting enables nab-paclitaxel to enhance reprogramming of M2-type macrophages into M1-like phenotype via ROS-HMGB1-TLR4 axis and inhibition of tumor growth and metastasis
Source: Theranostics. 2024 Apr 8;14(6):2605–21. doi: 10.7150/thno.92672 (PMC11024855; doi:10.7150/thno.92672)
Supplement: Supplementary file 1 — Supplementary methods and figures. [file thnov14p2605s1.pdf]

Supplementary Information

Supplementary Methods

Hemolysis assays

Mouse blood was collected in K2EDTA-coated tubes (BD, Franklin lakes, NJ). To isolate red blood cells (RBCs), 2 mL of the blood sample was added to 4 mL of Dulbecco's phosphate-buffered saline (D-PBS) and then centrifugated at 10,000 × g for 5 min. The RBCs were further washed five times with 10 mL of D-PBS and finally diluted to 20 mL with D-PBS. The 0.2 mL of diluted RBC suspension was exposed to 0.8 mL of IL4RPep-1, Abx, Ctrl-Abx, and IL4R-Abx in D-PBS to make the working concentrations of 2, 10, 50, 100, 200, and 400 μg/mL. Distilled water (positive group), and D-PBS (negative group) were used as control. After incubation at room temperature for 4 h and centrifugation for 5 min at 10,016 × g, 100 μL of the supernatant were transferred to a well of 96-well plates and the absorbance was measured by a microplate reader at 577 nm with the 655 nm as a reference. The hemolytic degree was expressed by the hemolytic ratio as the following formula: hemolysis ratio = (OD(test) − OD(negative control))/(OD(positive control) − OD(negative control)) × 100%.

Western blot analysis

M2 macrophages were lysed, and membrane proteins were enriched using M-PER mammalian protein extraction reagent (Thermo Fisher, Rockford, IL). Samples were electrophoresed in 10% polyacrylamide gel and transferred to a polyvinylidene difluoride membrane. The membrane was blocked with 5% skimmed milk in Tris-buffered saline containing 0.1% Tween-20 and then

incubated with antibodies against iNOS (Abcam, Cambridge, UK) and Arg-1 (Abcam) at 4 ºC overnight followed by incubation with a horse radish peroxidase-conjugated secondary antibody (Cell Signaling Technology, Danvers, MA) at room temperature for 1 h. The membrane was incubated with a substrate (Thermo Fisher) and then with a chemiluminescence detection reagent (FujiFilm, Tokyo, Japan). The protein band intensity was quantified using ImageJ software (National Institute of Health, Bethesda, MD).

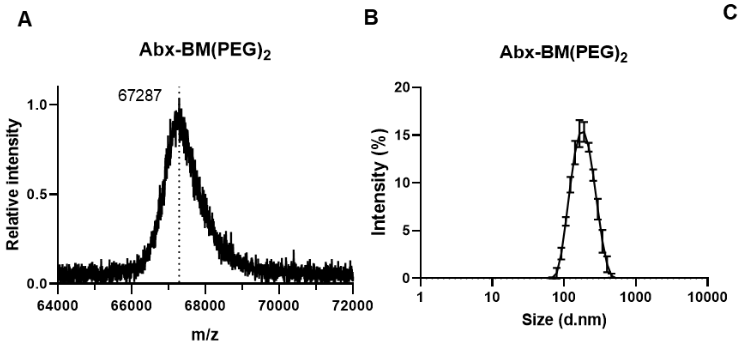

C

	Z-average	PDI
**Abx**	160.1±0.802	0.146±0.013
**Abx-BM(PEG)$_2$**	169.5±2.281	0.135±0.005
**IL4R-Abx**	161.8±1.716	0.154±0.018

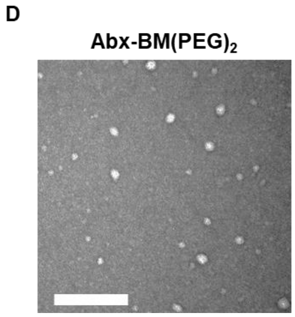

Figure S1. Characterization of Abx, Abx-BM(PEG)2, and IL4R-Abx. **(A)** MALDI-TOF-MS spectra of Abx-BM(PEG)$_2$. Shown are data from 64 kDa to 72 kDa. **(B)** Hydrodynamic size distribution of Abx-BM(PEG)$_2$ measured by DLS. **(C)** Z-average and polydispersity index (PDI) of Abx, Abx-BM(PEG)2, and IL4R-Abx measured by DLS. All groups were dissolved at 10 mg/mL in PBS buffer. **(D)** TEM images of Abx-BM(PEG)$_2$ dissolved at 0.01 mg/mL in deionized water.

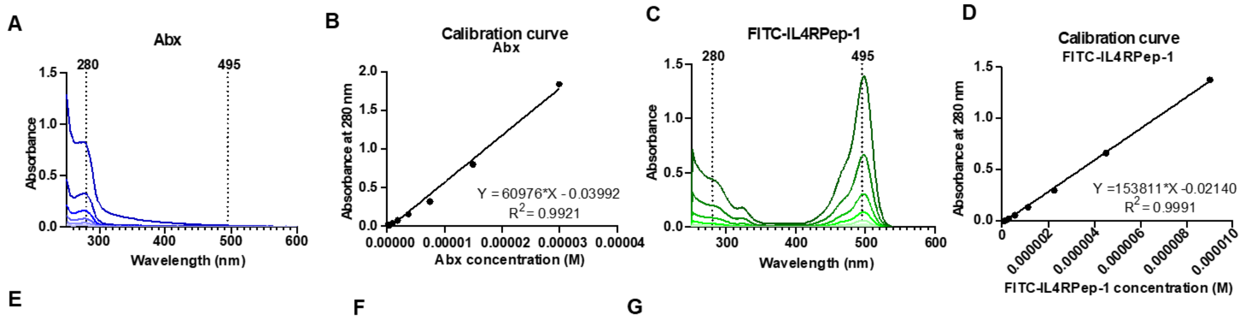

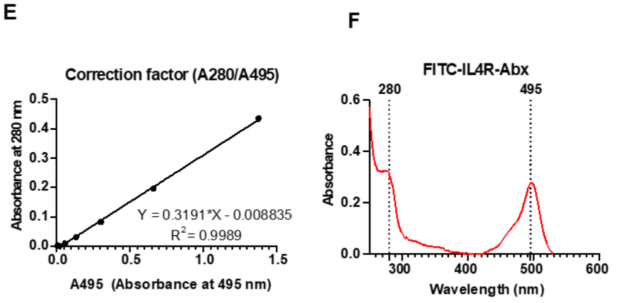

G

MW_{Alb}	MW_{FI}	Average MW_{abx}	Mol% Alb in Abx (%)	ε_{Abx} ($M^{-1}cm^{-1}$)	ε_{FI} ($M^{-1}cm^{-1}$)
66348	1665.1	7646.1	10.37	60975	153803

	FITC-IL4R-Abx solution (M)				
CF	A280	A495	[Abx] (M)	[Alb] (M)	**Conjugation ratio ([FI]/[Alb])**
0.3191	0.3166	0.2782	2.922×10^{-5}	3.030×10^{-6}	**0.5969**

Figure S2. Quantification of the degree of FITC-IL4RPep-1 conjugation to IL4R-Abx. **(A)** UV-Vis absorbance spectrum of Abx, displaying the maximum absorbance peak at 280 nm (λ_{max}). **(B)** Standard calibration curve using absorbance at 280 nm (A280) to determine the concentration of Abx. The extinction coefficient (ε_{Abx}) is calculated to be 60,976 $M^{-1}cm^{-1}$. **(C)** UV-Vis absorbance spectrum of FITC-IL4RPep-1 (FI), displaying the maximum absorbance peak at 495 nm (λ_{max}). **(D)** Standard calibration curve using absorbance at 495 nm (A495) to determine the concentration of FI. The extinction coefficient (ε_{FI}) is calculated to be 15,3811 $M^{-1}cm^{-1}$. **(E)** To compensate the A280 from FI, the correction factor (CF) is calculated to be 0.3191 by dividing the A280 of FIto the A495 of FI. The slope represents the CF. **(F)** UV-Vis absorbance spectrum of FITC-IL4R-Abx. **(G)** Constant values used to calculate the conjugation ratio of FI to albumin. Average Abraxane MW (g/mol) = $1/(0.9/MW_{Alb}+0.1/MW_{PTX})$; Mol% Alb in Abx (%) = $(0.9/MW_{Alb})/(0.9/MW_{Alb}+0.1/MW_{PTX}) \times 100$; [Alb] = [Abx] × Mol% Alb in Abx/100; [Abx] = (A280-(A495 × CF))/ε_{Abx}; conjugation ratio ([FI]/[Alb]) = A495/ (ε_{FI} × [Alb]).

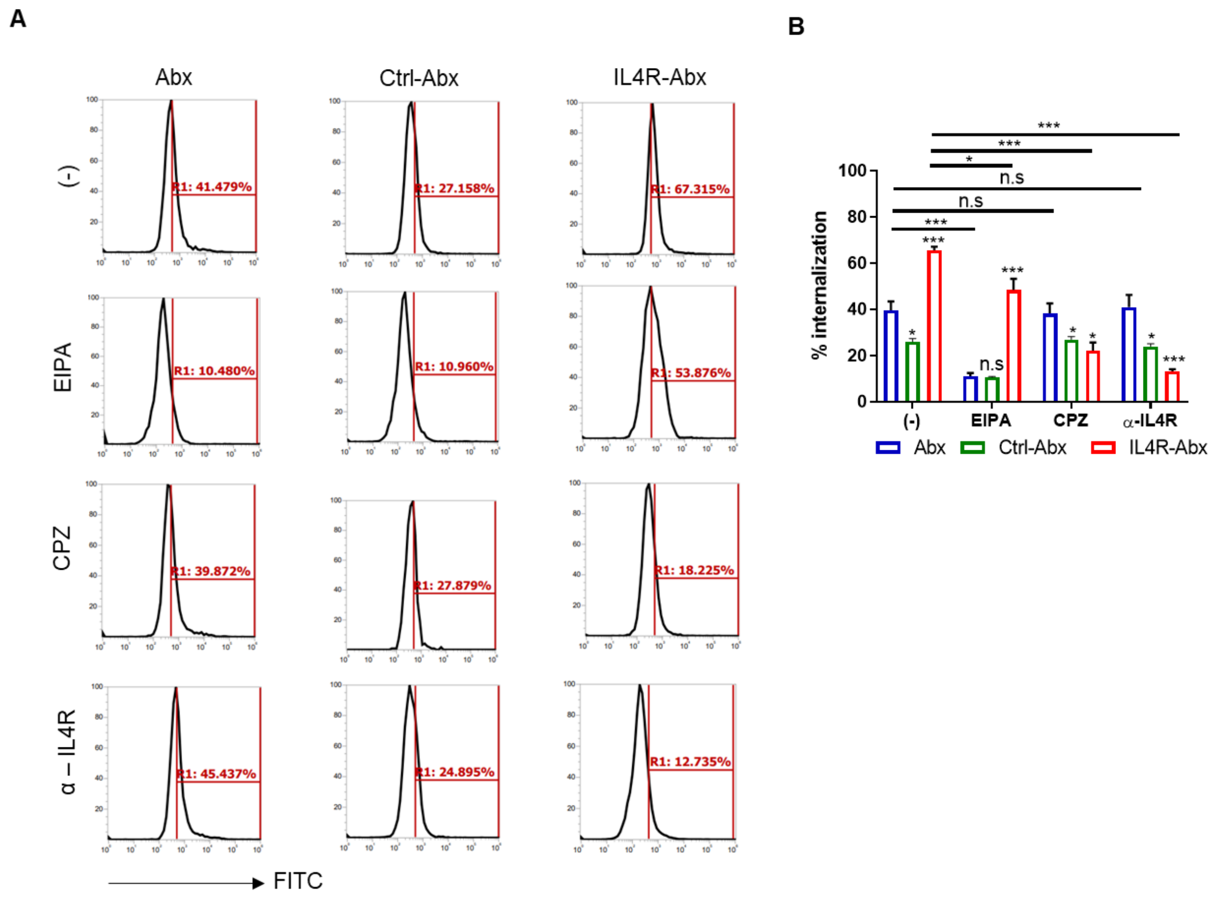

Figure S3. Internalization of Abx, Ctrl-Abx, and IL4R-Abx into M2 macrophages. **(A)** M2 macrophages were incubated using 5 μg/mL of fluorescein isothiocyanate (FITC) dye (green)-labeled Abx, Ctrl-Abx, and IL4R-Abx for 2 h with or without pre-treatment with 5-(n-ethyl-n-isopropyl) amiloride (EIPA) (50 μM), chlorpromazine (CPZ) (50 μM), and anti-IL4R antibody (100 μM) for 30 min. After incubation, the internalization was analyzed using a flow cytometer. **(B)** The percent internalization was quantified. Data are expressed as mean ± standard deviation (SD) in the three separate experiments. *P* values of Ctrl-Abx and IL4R-Abx compared to Abx in each group are shown on the top of bars. *, $P < 0.05$; **, $P < 0.01$; ***, $P < 0.001$; n.s., not significant by one-way ANOVA followed by Tukey's multiple post hoc test.

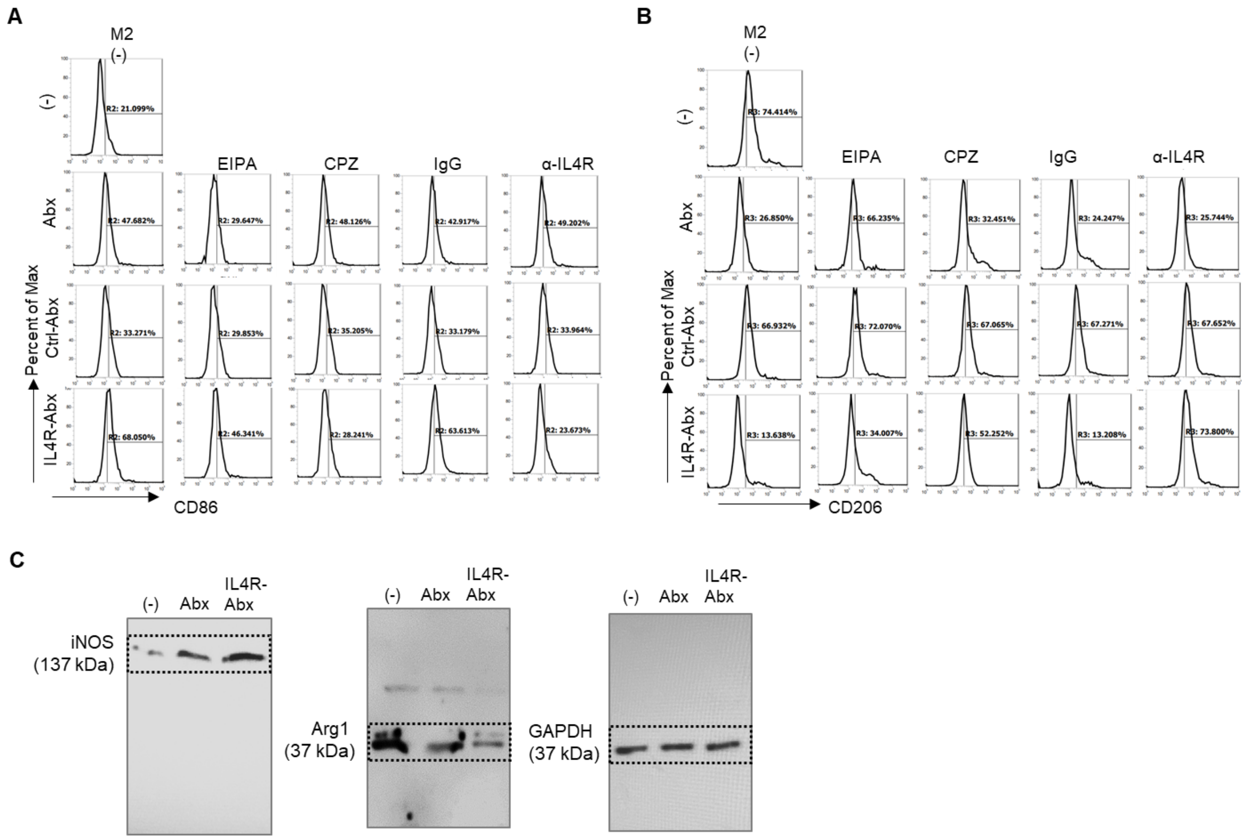

Figure S4. Reprogramming of M2 macrophages into M1-like phenotype by IL4R-Abx. **(A)** M2 macrophages were incubated with 5 μg/mL of Abx and IL4R-Abx for 24 h. The cell lysates were subjected to western blot analysis using antibodies against iNOS and Arg1. Glyceraldehyde-3-phosphate dehydrogenase (GAPDH) was used as control. **(B, C)** M2 macrophages were incubated with 5 μg/mL of Abx, Ctrl-Abx, and IL4R-Abx for 24 h with or without pre-treatment with EIPA (50 μM), CPZ (50 μM), anti-IL4Rα antibody (100 μM), and IgG (100 μM) for 30 min. Shown are representative histograms of the expression of CD86 **(B)** and CD206 **(C)**.

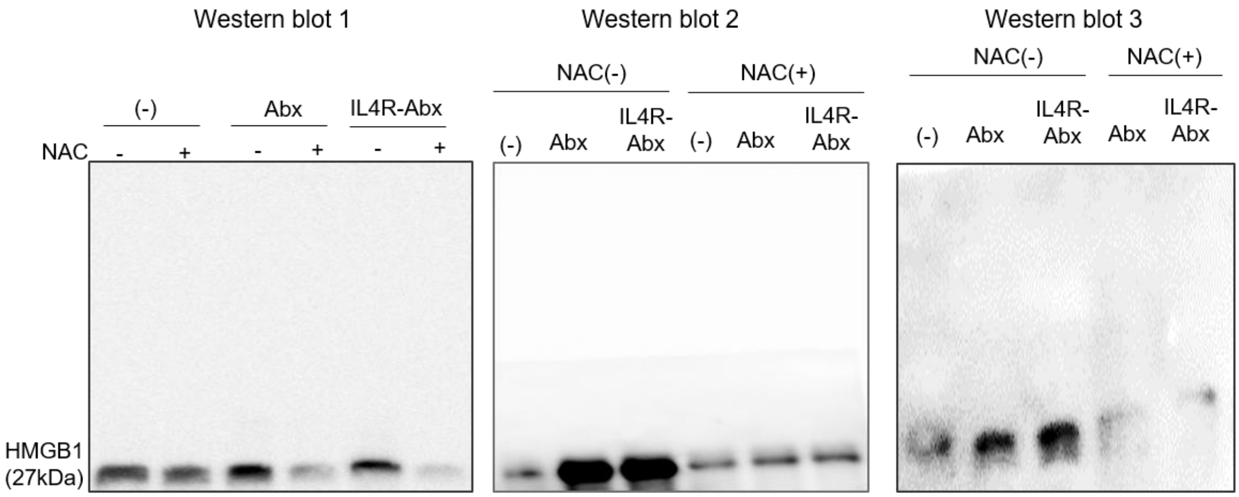

Figure S5. IL4R-Abx increases the extracellular release of HMGB1 into the culture medium via ROS upregulation. M2 macrophages were incubated with 5 μg/mL of Abx and IL4R-Abx for 6 h with or without pre-treatment with 50 mM of N-acetyl-l-cysteine (NAC) for 30 min. The conditioned medium was collected and subjected to western blotting using an anti-HMGB1 antibody. Shown are the results of three individual experiments.

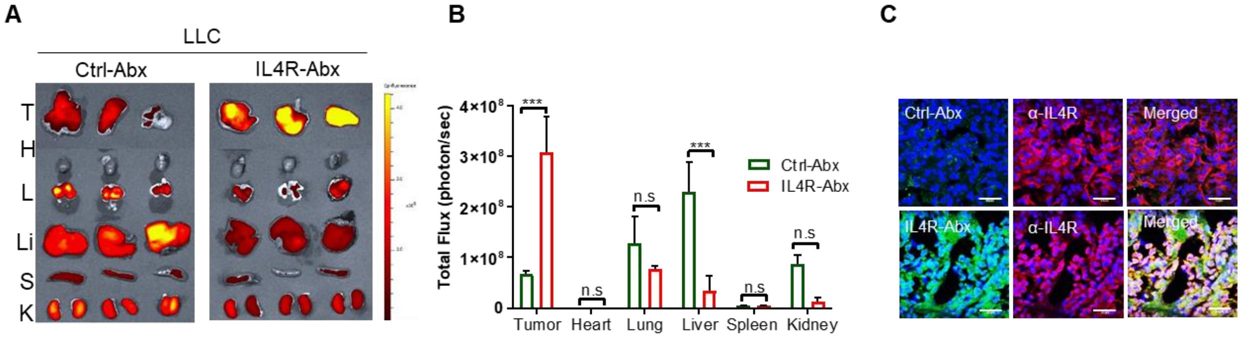

Figure S6. Tumor homing of IL4R-Abx in mice bearing LLC mouse lung tumor. **(A, B)** *Ex vivo* imaging of tumor and organs isolated at 6 h after injection of Ctrl-Abx and IL4R-Abx (5 mg/kg body weight) into mice bearing LLC lung tumor **(A)** and quantification of the total flux (photon/s) of each organ **(B)**. T, tumor; H, heart; L, lung; Li, liver; S, spleen; K, kidney. Circles (cyan blue) suggest tumor nodules in the lungs. **(C)** Histochemical analysis. Tumor tissues isolated at 6 h after injection of FITC dye (green)-labeled Ctrl-Abx and IL4R-Abx. Tissues were incubated using an anti-IL4R antibody (red) and with DAPI for nucleus staining (blue), and images were merged. Scale bars, 20 μm. Data are expressed as mean ± SD. ***, $P < 0.001$; n.s., not significant by two-way ANOVA followed by Bonferroni post hoc test (n =3 per group).

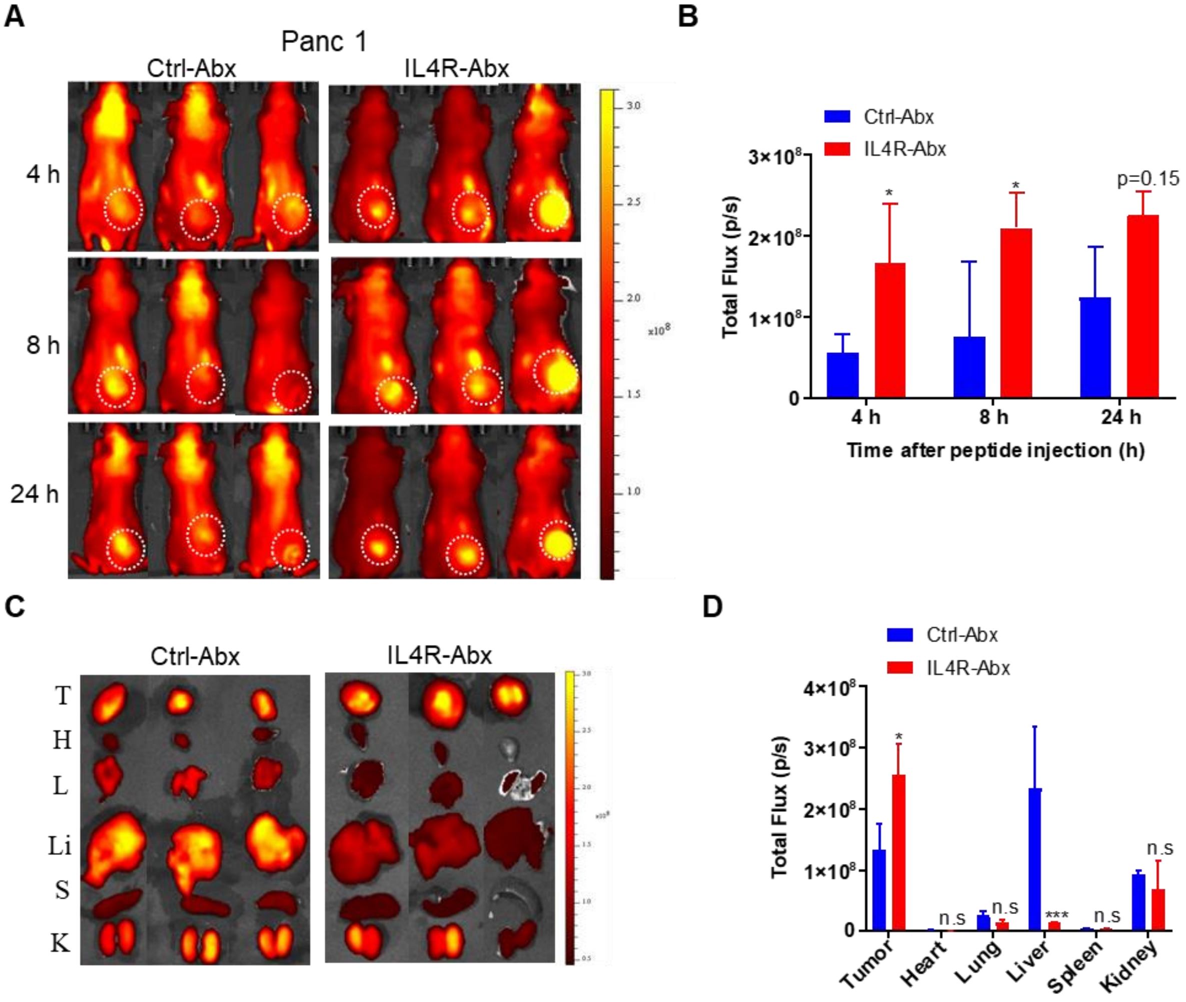

Figure S7. Tumor homing of IL4R-Abx in mice bearing Panc1 human pancreatic tumor. **(A)** Whole-body fluorescence imaging of mice bearing Panc1 tumor at 4 h, 8 h and 24 h after injection of near-infrared fluorescence dye-labeled Ctrl-Abx and IL4R-Abx (5 mg/kg body weight). Dotted circles represent the tumor site. **(B)** Total flux quantification (photon/s) of the tumor site in (A). **(C, D)** *Ex vivo* imaging of tumor and organs isolated after the whole-body imaging **(C)** and total flux quantification (photon/s) of each organ **(D)**. T, tumor; H, heart; L, lung; Li, liver; S, spleen; K, kidney. Data are expressed as mean ± SD. *, $P < 0.05$; ***, $P < 0.001$; n.s., not significant

compared to Ctrl-Abx by two-way ANOVA followed by Bonferroni post hoc test (n = 3 per group).

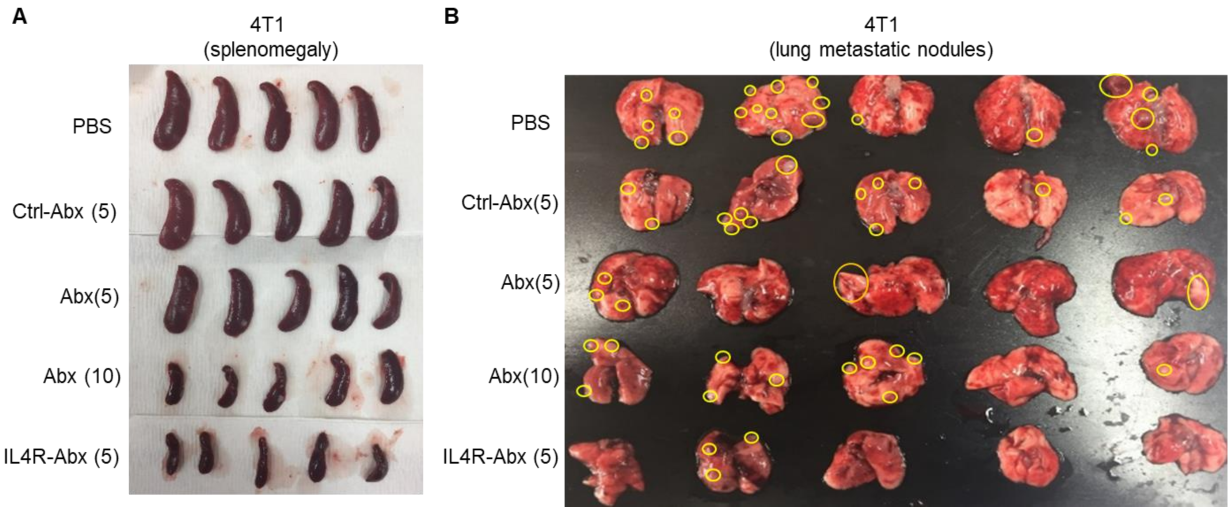

Figure S8. Inhibition of splenomegaly and lung metastasis by IL4R-Abx in mice bearing 4T1 mouse breast tumor. Mice bearing 4T1 tumor were injected with Abx, Ctrl-Abx, and IL4R-Abx (5 or 10 mg/kg body weight, weekly for 4 weeks). Photos represent spleens **(A)** and lungs **(B)** isolated from tumor-bearing mice at the end of treatments. The number of metastatic nodules in lungs circled in yellow color were counted.

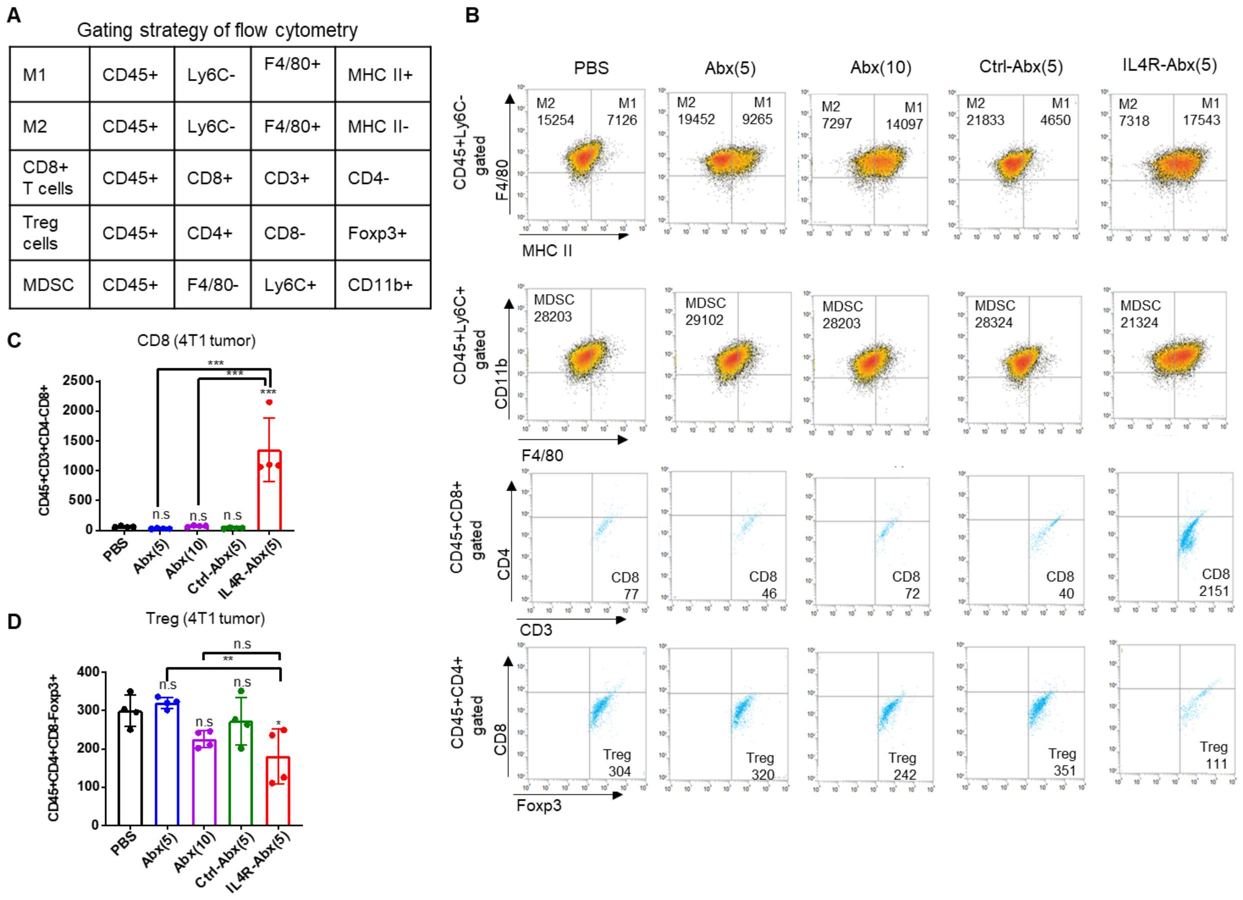

Figure S9. Immune cell analysis of 4T1 tumor tissues after IL4R-Abx treatment. **(A)** Mice bearing 4T1 tumor were injected with Abx, Ctrl-Abx, and IL4R-Abx (5 or 10 mg/kg body weight, weekly for 4 weeks). At the culmination of the treatments, tumors were isolated for immune cell analysis using the gating strategy for flow cytometry. **(B)** Representative histograms showing the population of M1-macrophages, M2-macrophages, MDSCs, CD8+ T cells, and Tregs after treatments. **(C)** CD45+CD3+CD4-CD8+ T cell population. **(D)** CD45+CD4+CD8-Foxp3+ Treg population. Data are expressed as mean ± SD. *P* values compared to saline-treated group are shown on the top of bars. *, $P < 0.05$; **, $P < 0.01$; ***, $P < 0.001$; n.s., not significant by one-way ANOVA followed by Tukey's multiple post hoc test ($n = 4$ per group).

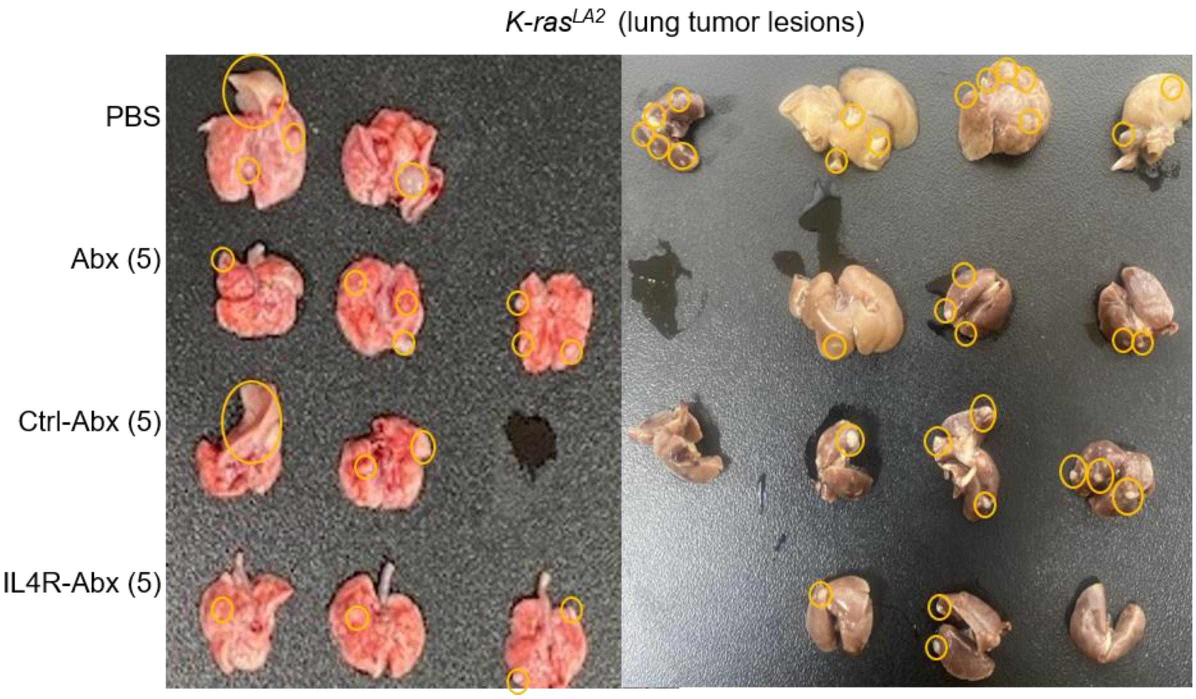

Figure S10. Therapeutic efficacy of IL4R-Abx in mice bearing *K-ras*^{LA2} mutant tumor. Mice bearing *K-ras*^{LA2} tumor were injected with Abx, Ctrl-Abx, and IL4R-Abx (5 mg/kg body weight, weekly for 4 weeks). Photos represent lungs isolated from tumor-bearing mice at the end of treatments. The number of tumor lesions (circled in yellow color) were counted.

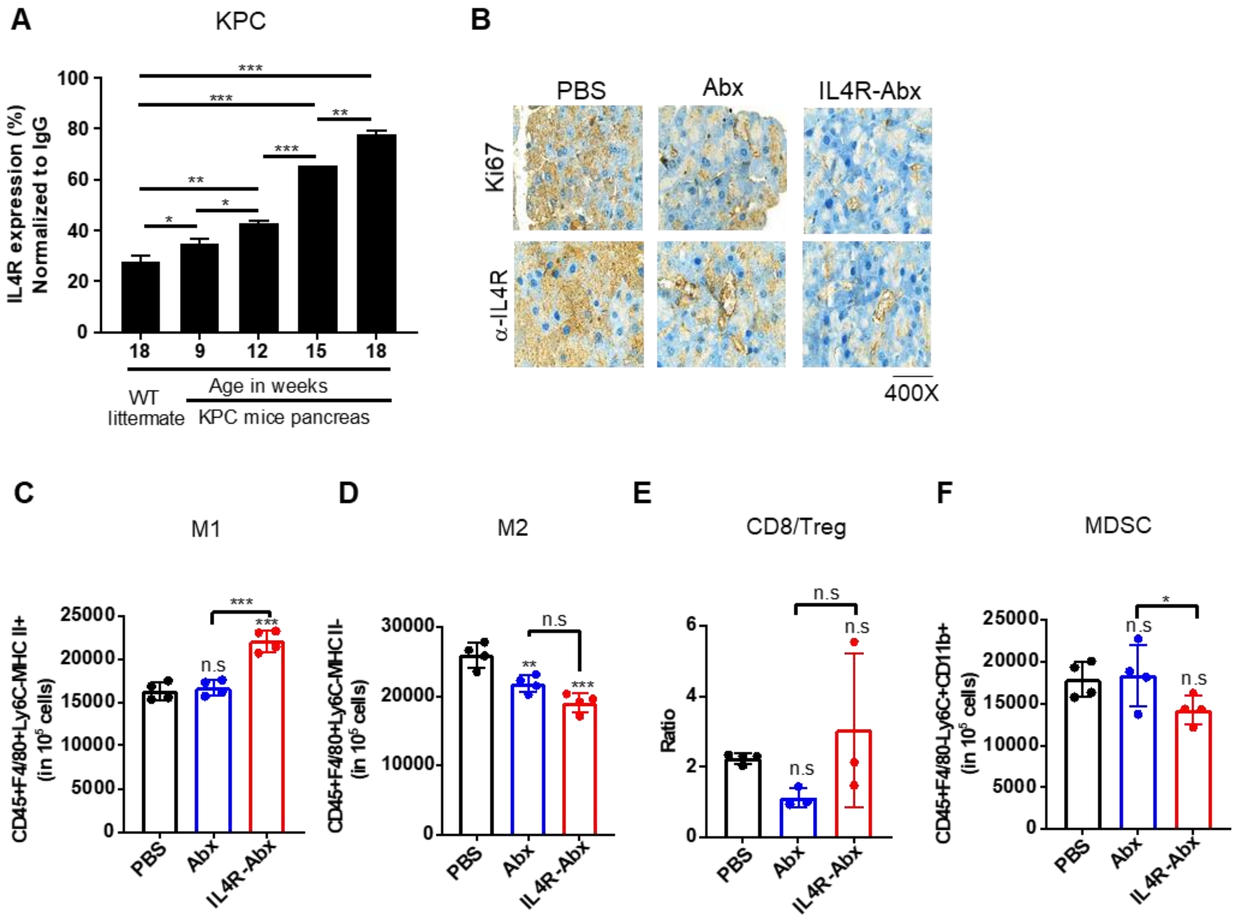

Figure S11. Tumor growth inhibition and antitumor immunity improvement by IL4R-Abx in KPC pancreatic tumor mice. **(A)** Immunohistochemical analysis of IL4R expression in the pancreas of KPC (*LSL-Kras*$^{G12D/+}$*LSL-p53*$^{R172H/+}$*PDX-1 Cre*) transgenic tumor mice at different ages compared to WT littermate. Tissue sections were stained with anti-IL4R antibody, and the intensity was measured using ImageJ software. **(B-F)** KPC mice were treated with Abx and IL4R-Abx (5 mg/kg body weight, weekly for 4 weeks). At the culmination of the treatments, tissue sections were prepared and stained with anti-Ki67 and anti-IL4R antibodies and then counter-stained with hematoxylin **(B)**. Cell suspension of tumor tissues were prepared and subjected to flow cytometry

for immune cell analysis in another set of experiment. The population of CD45+F4/80+Ly6C-MHC II+ M1 macrophages **(C)**, CD45+F4/80+Ly6C-MHC II- M2 macrophages **(D)**, CD8/Treg ratio **(E)**, and CD45+F4/80-Ly6C+CD11b+ MDSCs **(F)** in 10^5 cells of tumor tissues were determined. Data are expressed as mean ± SD. *P* values compared to saline-treated group are shown on the top of bars. *, $P < 0.05$; **, $P < 0.01$; ***, $P < 0.001$; n.s., not significant by one-way ANOVA followed by Tukey's multiple post hoc test ($n = 3$ per group).

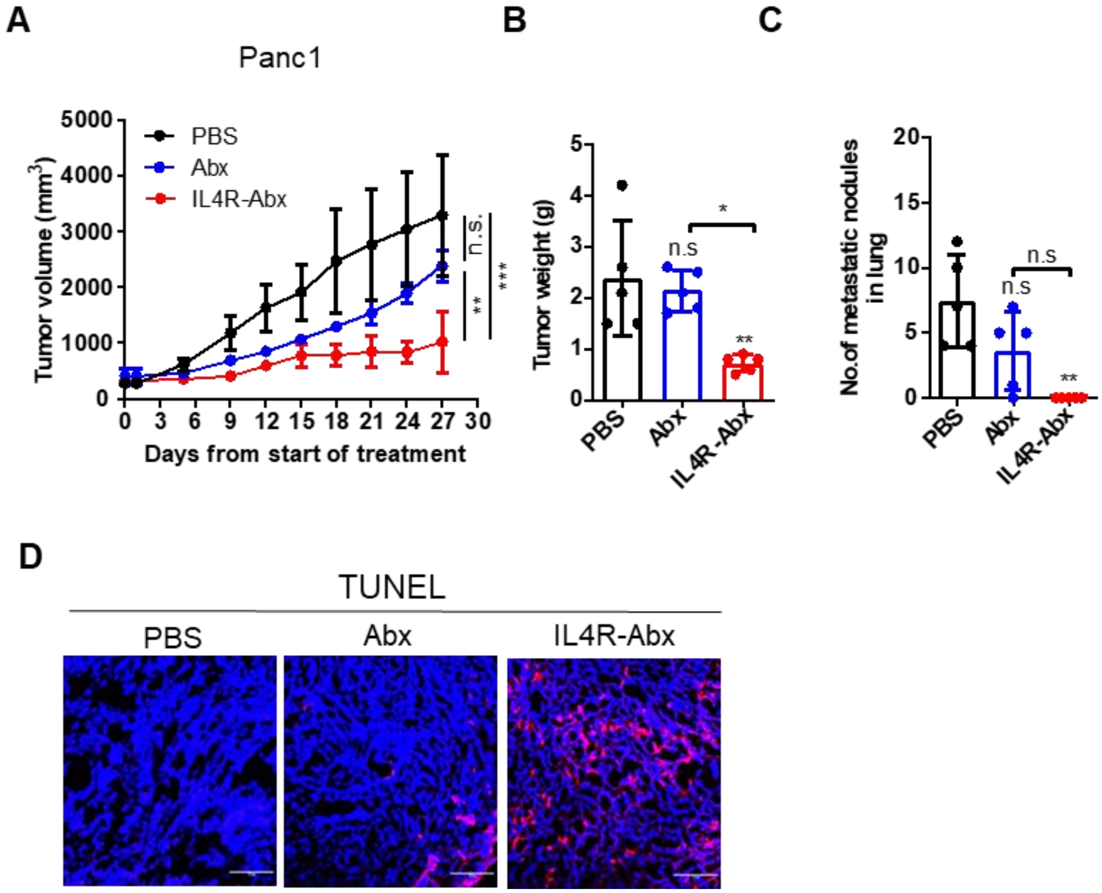

Figure S12. Tumor growth and metastasis inhibition by IL4R-Abx in mice bearing Panc-1 human pancreatic tumor xenografts. **(A-C)** Mice bearing Panc-1 pancreatic tumor were intravenously injected with Abx and IL4R-Abx (5 mg/kg of body weight, weekly for 4 weeks). At the treatment culmination, the tumor volume **(A)**, tumor weight **(B)**, and the number of metastatic nodules in the lungs **(C)** were determined. Data are expressed as mean ± SD. *P* values compared to the saline-treated group are shown on the top of bars. *, $P < 0.05$; **, $P < 0.01$; ***, $P < 0.001$; n.s., not significant by one-way ANOVA followed by Tukey's multiple post hoc test ($n = 5$ per group). **(D)** Tissues sections were prepared and subjected to TUNEL assay. Nucleus was counter-stained with DAPI. Scale bars, 20 μm.

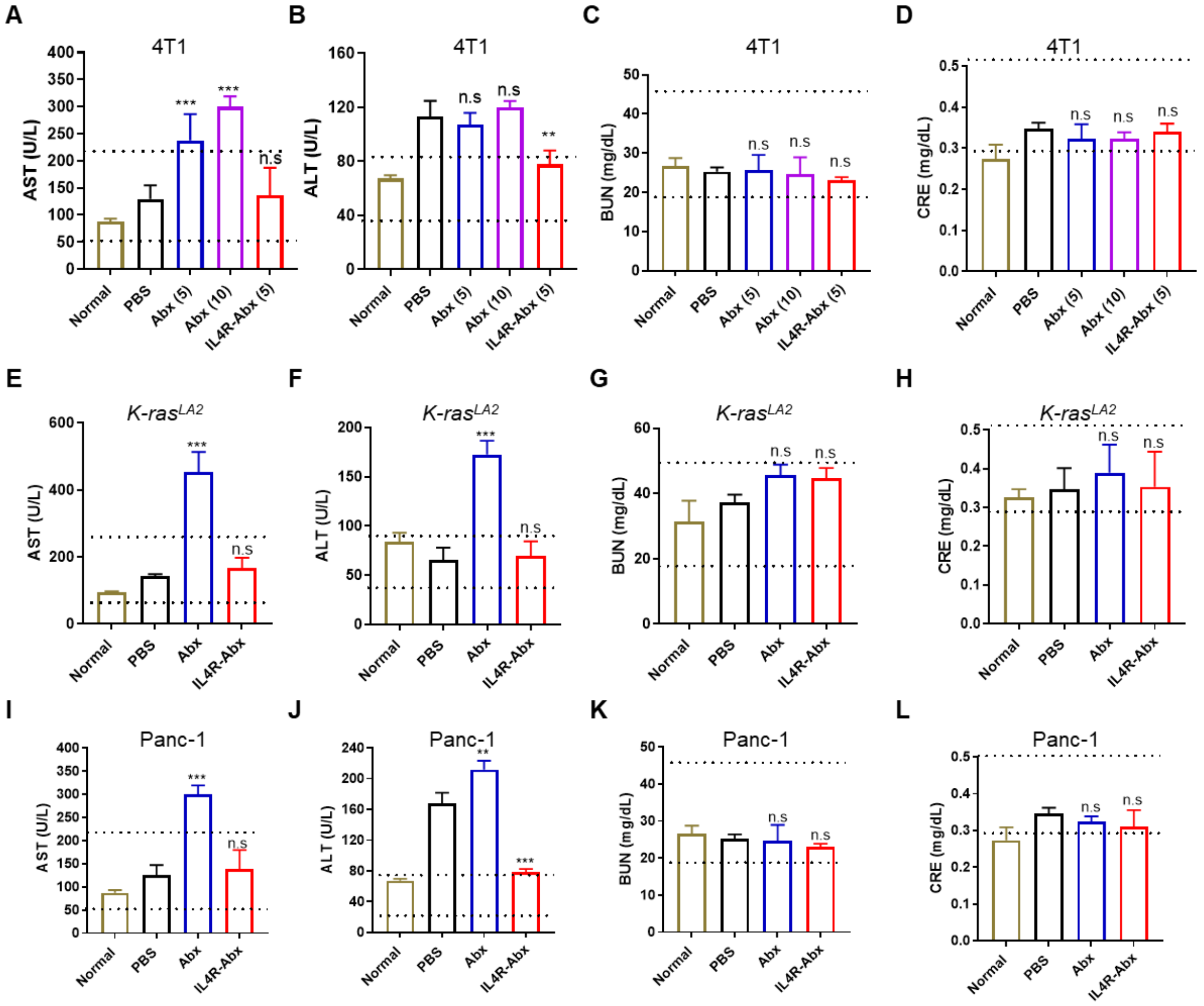

Figure S13. Systemic side effects of IL4R-Abx on the liver and kidney function. Mice bearing tumor were injected with Abx and IL4R-Abx (5 or 10 mg/kg body weight, weekly for 4 weeks). At the end of the treatments, blood was collected and analysis of serum levels of aspartate aminotransferase (AST), alanine aminotransferase (ALT), blood urea nitrogen (BUN), and creatinine (CRE) was conducted in 4T1 tumor mice **(A-D)**, *K-ras*^{LA2} mutant mice **(E-H)**, and Panc-1 tumor mice **(I-L)**. Dotted lines indicate the normal range of each parameter. Data are expressed

as mean ± SD. *, $P < 0.05$; **, $P < 0.01$; ***, $P < 0.001$; n.s., not significant by one-way ANOVA followed by Tukey’s multiple post hoc test ($n = 6$ per group).

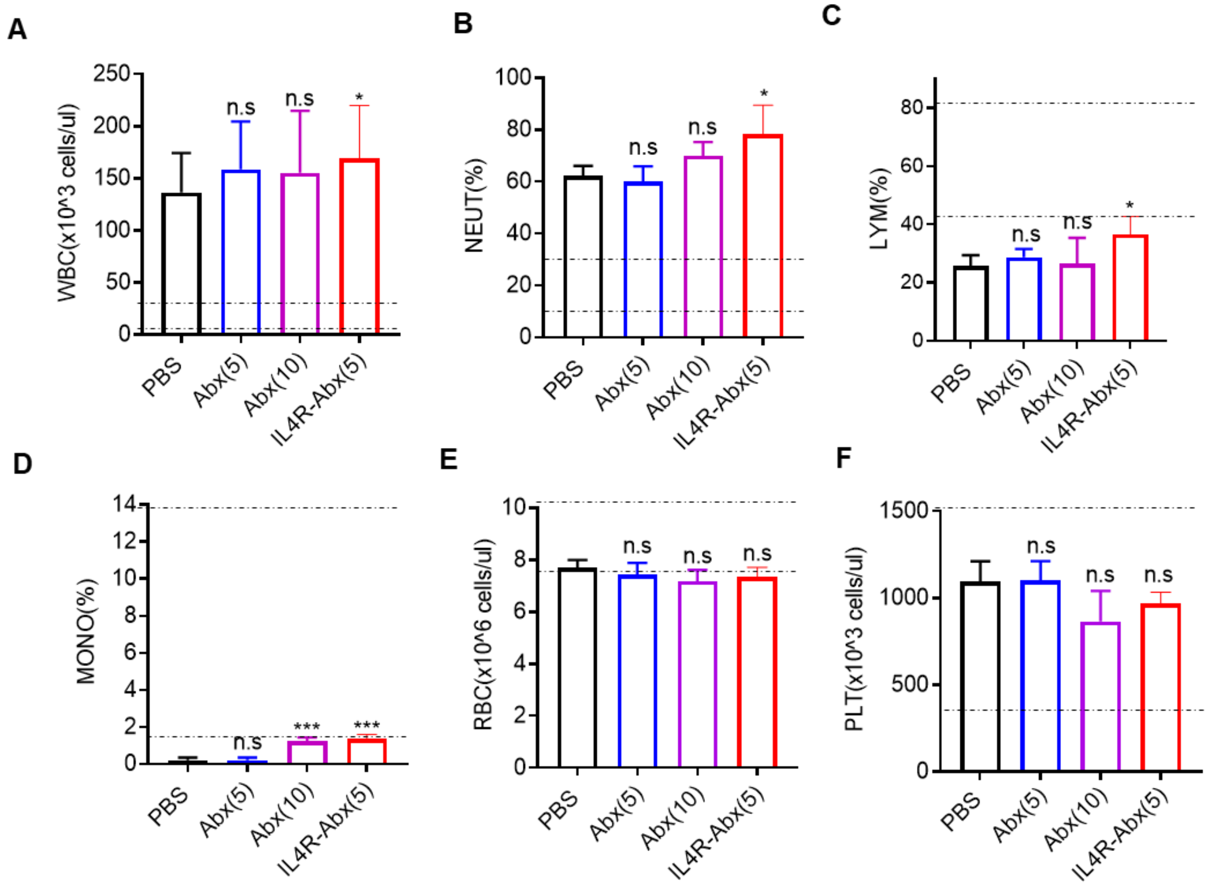

Figure S14. Effects of IL4R-Abx on hematological parameters. Mice bearing 4T1 tumor were injected with Abx and IL4R-Abx (5 or 10 mg/kg body weight, weekly for 4 weeks). At the end of the treatments, blood was collected and analyzed for the population of white blood cell (WBC) (**A**), neutrophil (NEUT) (**B**), lymphocyte (LYM) (**C**), monocyte (MONO) (**D**), red blood cell (RBC) (**E**), and platelet (PLT) (**F**). Dotted lines indicate the normal range of each parameter. Data are expressed as mean ± SD. *, $P < 0.05$; **, $P < 0.01$; ***, $P < 0.001$; n.s., not significant by one-way ANOVA followed by Tukey's multiple post hoc test ($n = 6$ per group).

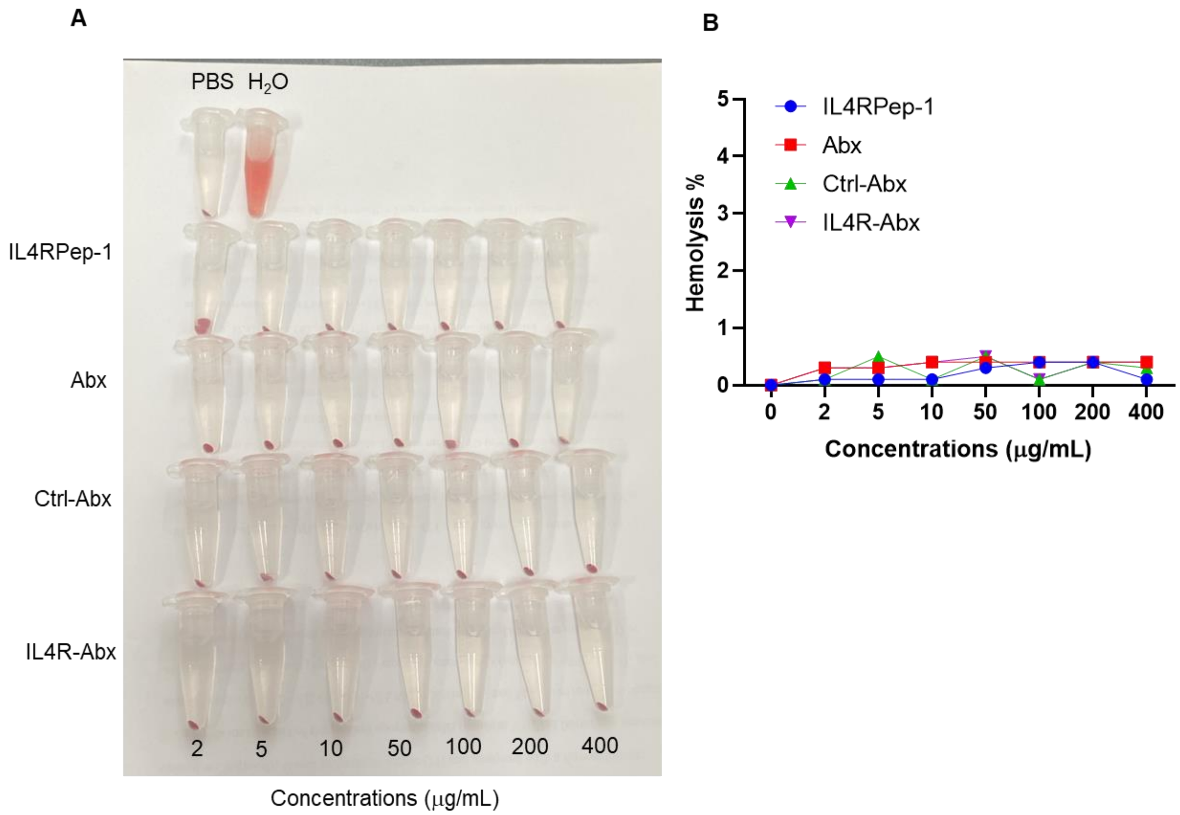

Figure S15. Hemolysis assays. (**A**) Mouse RBCs were collected and incubated with the indicated concentrations of IL4RPep-1, Abx, Ctrl-Abx, and IL4R-Abx for 4 h. Phosphate-buffered saline (PBS) and H_2O were used as negative and positive controls, respectively. (**B**) Percent hemolysis after incubation with the indicated reagents.

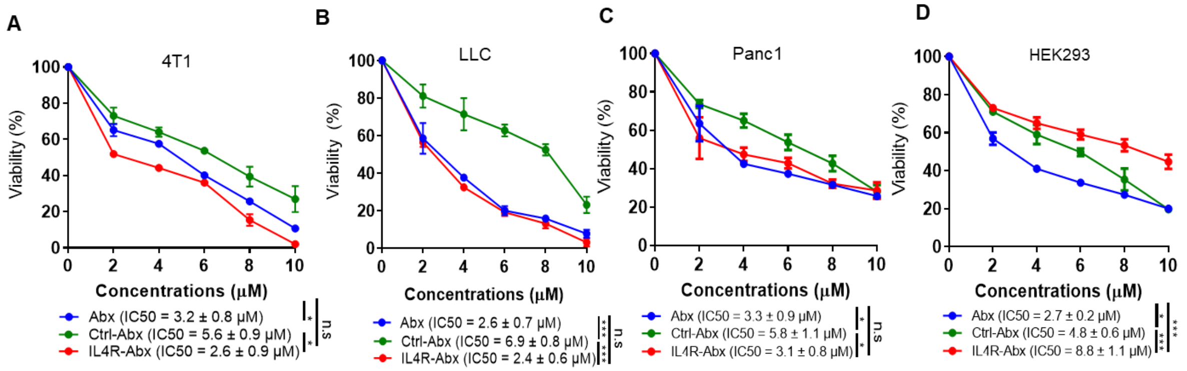

Figure S16. IL4R-Abx cytotoxicity in tumor and normal cells. 4T1 **(A)**, LLC **(B)**, and Panc-1 **(C)** tumor cells and HEK293 normal cells **(D)** were treated with the indicated concentrations of Abx, Ctrl-Abx, and IL4R-Abx for 24 h and subjected to cytotoxicity assays. Data are expressed as mean ± SD in the three separate experiments. *, $P < 0.05$; **, $P < 0.01$; ***, $P < 0.001$; n.s., not significant by one-way ANOVA followed by Tukey's multiple post hoc test.

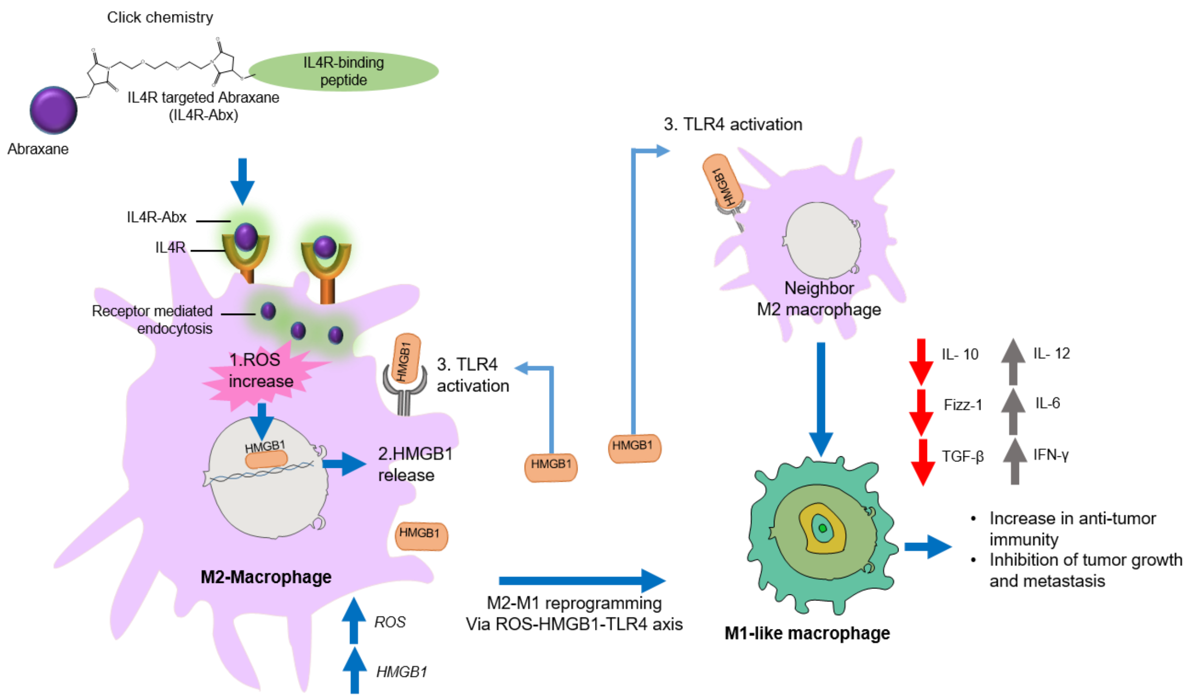

Figure S17. Schemes of the reprogramming of M2-macrophages into M1-like phenotype by IL4R-targeted Abx via ROS-HMGB1-TLR4 axis. Abraxane was conjugated with an IL4R-binding peptide using click chemistry. The IL4R-targeted abraxane (IL4R-Abx) was preferentially internalized into M2-macrophages via the receptor-mediated endocytosis and upregulates intracellular ROS levels. The increase in ROS induced nuclear-cytoplasmic translocation and extracellular release of HMGB1. The extracellular HMGB1 binds to and activates TLR4 on the surface of M2-macrophages. Activation of TLR4 decreases M2-macrophage markers (IL10, Fizz-1, and TGF-β) while increasing M1-macrophage markers (IL12, IL6, and IFN-γ) and skews M2-macrophages into M1-like phenotype. The M2 to M1 macrophage reprogramming contributes to the increase in anti-tumor immunity in the tumor microenvironment and inhibition of tumor growth and metastasis.
